# Supplementary material for: Palladium-Catalyzed β-C(sp3)–H Bond Arylation of Tertiary Aldehydes Facilitated by 2-Pyridone Ligands
Source: Molecules. 2024 Jan 3;29(1):259. doi: 10.3390/molecules29010259 (PMC10780448; doi:10.3390/molecules29010259)
Supplement: Supplementary file 1 [file molecules-29-00259-s001.zip › molecules-2766432-supplementary.pdf]

Supplementary Materials

Palladium-catalyzed β-C(sp^3)–H bond arylation of tertiary aldehydes facilitated by 2-pyridone ligands

Ziting Xu [1], Zhi Li [1], Chong Liu [2], Ke Yang [1,*] and Haibo Ge [2,*]

[1] Jiangsu Key Laboratory of Advanced Catalytic Materials & Technology, School of Petrochemical Engineering, Changzhou University, Changzhou, Jiangsu 213164, China
[2] Department of Chemistry and Biochemistry, Texas Tech University, Lubbock, Texas 79409, USA
* Correspondence: keyang@cczu.edu.cn (K.Y.); haibo.ge@ttu.edu (H.G.)

Table of contents

I. Starting materials··········S2

II. ^{1}H and ^{13}C NMR Spectra··········S2-S39

I. Starting materials

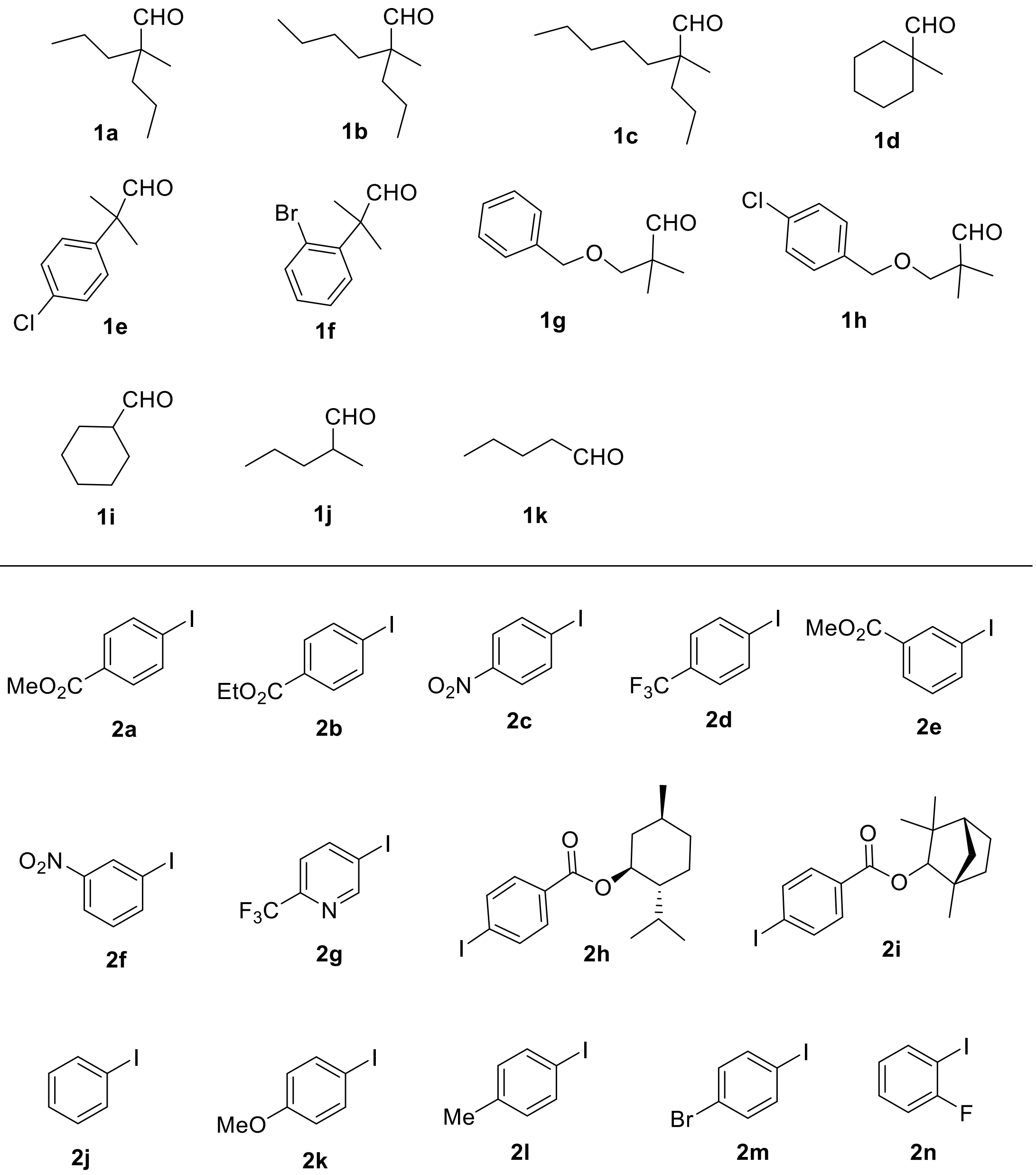

Figure S1. Aliphatic aldehydes and aryl iodides

II. ^{1}H and ^{13}C NMR Spectra

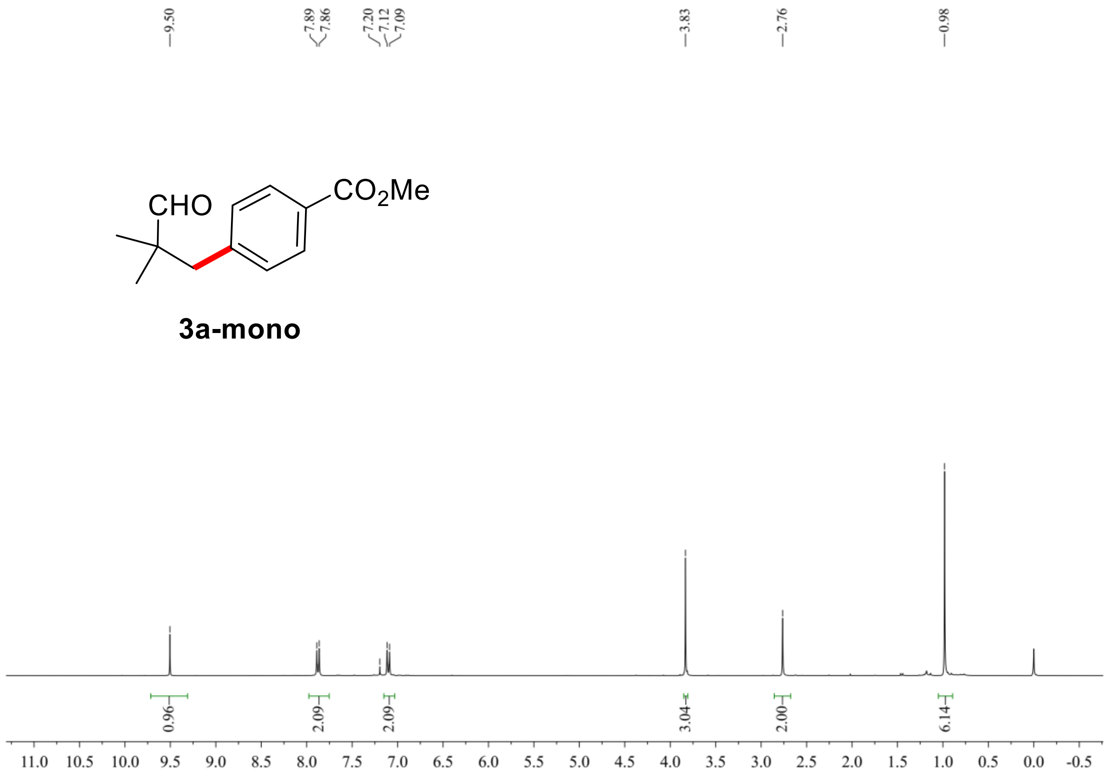

Figure S2 ^{1}H NMR spectrum (300MHz, $CDCl_3$, 298K) of **3a-mono**

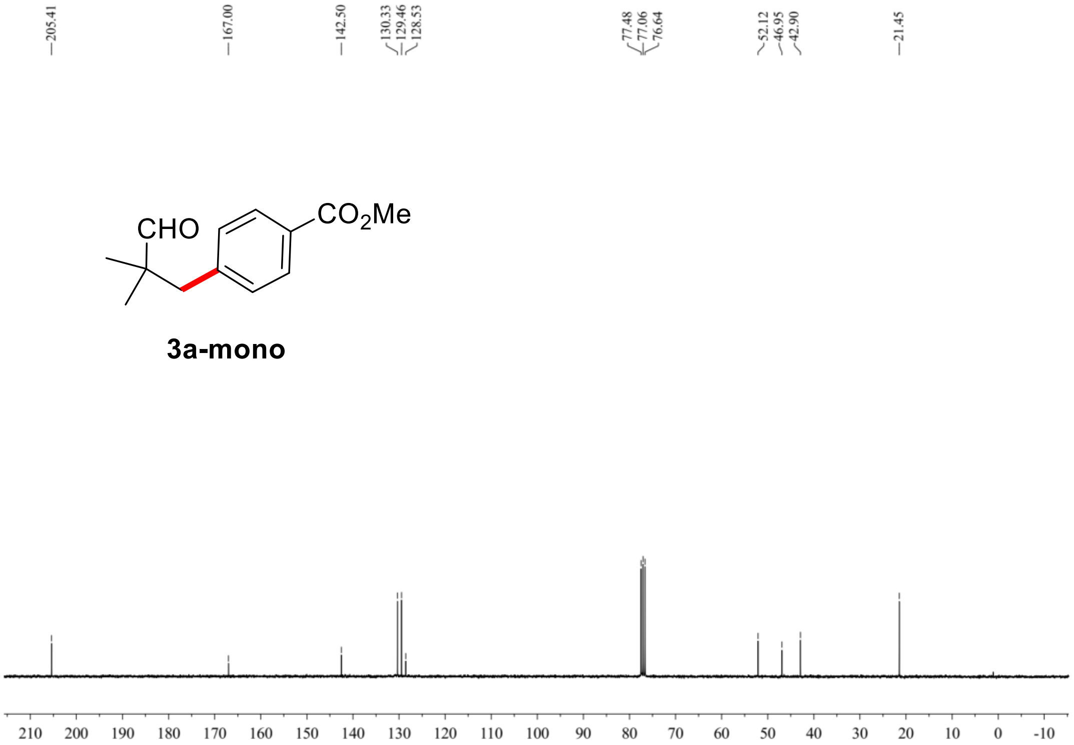

Figure S3 ^{13}C NMR spectrum (75MHz, $CDCl_3$, 298K) of **3a-mono**

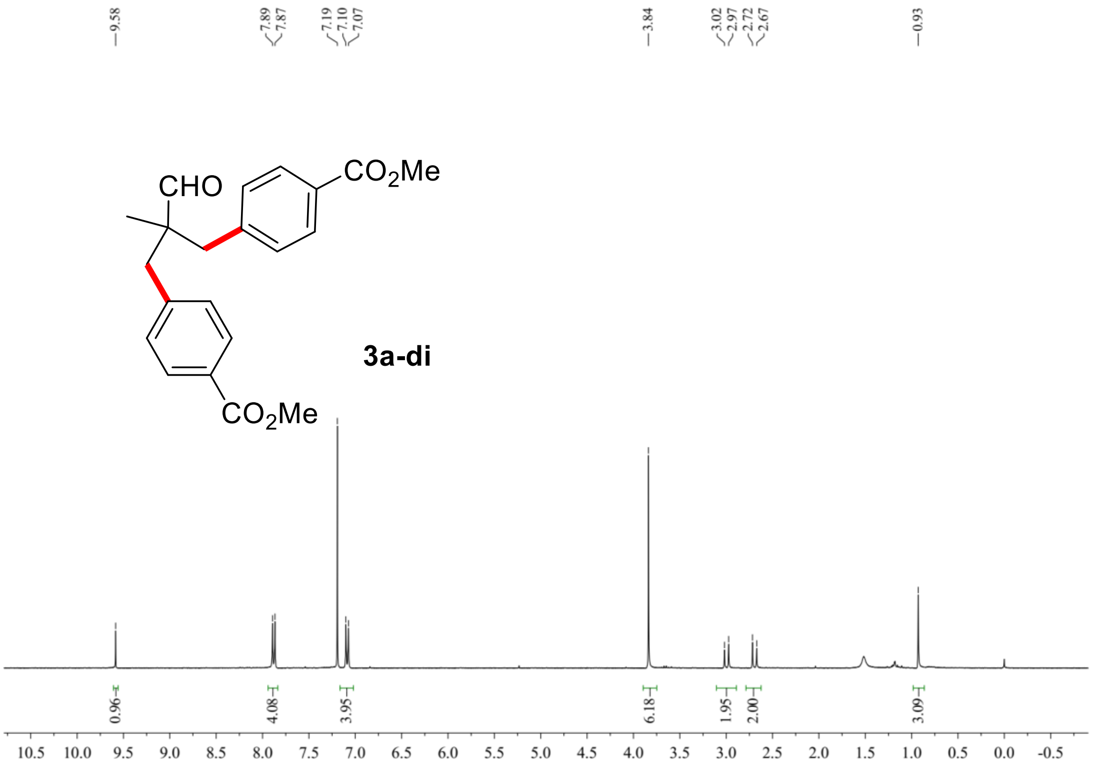

Figure S4 ^{1}H NMR spectrum (300MHz, $CDCl_3$, 298K) of **3a-di**

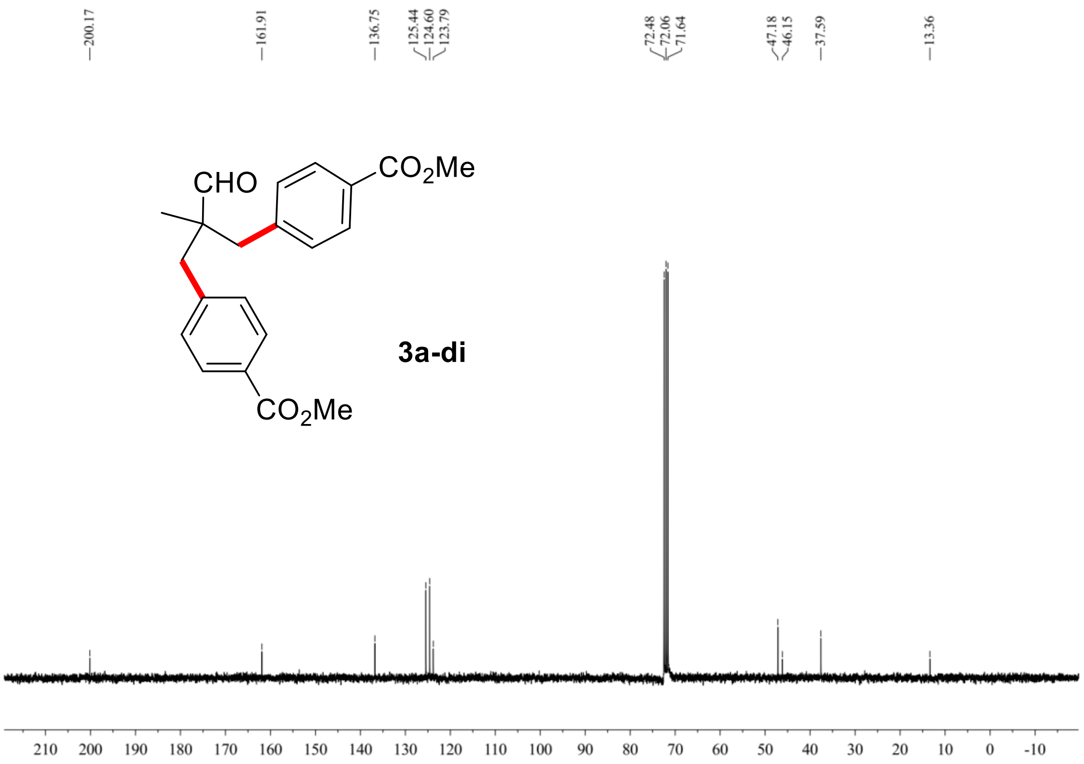

Figure S5 ^{13}C NMR spectrum (75MHz, $CDCl_3$, 298K) of **3a-di**

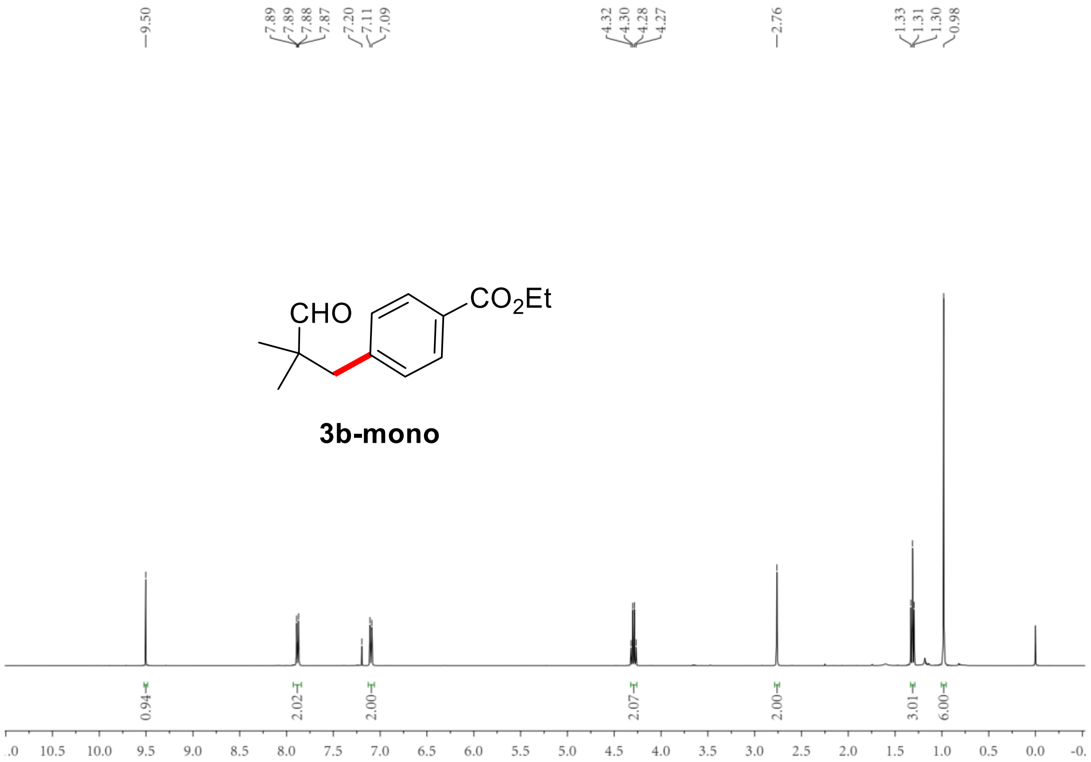

Figure S6 ^{1}H NMR spectrum (400MHz, $CDCl_3$, 298K) of **3b-mono**

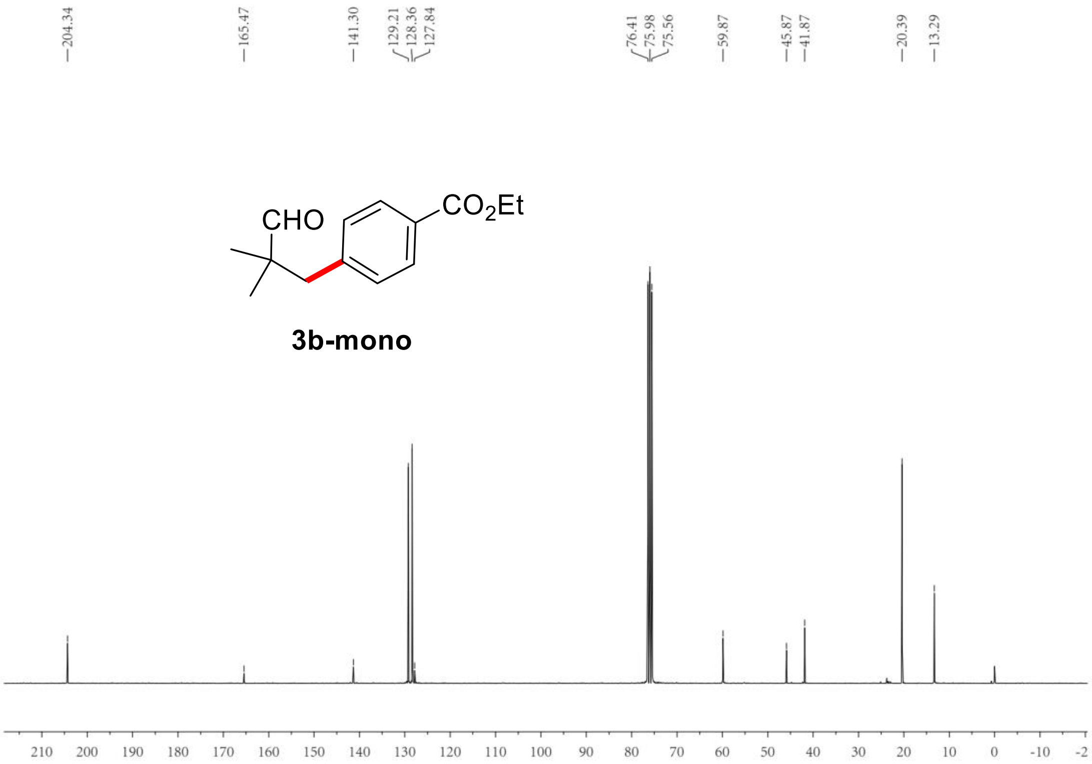

Figure S7 ^{13}C NMR spectrum (75MHz, $CDCl_3$, 298K) of 3**b-mono**

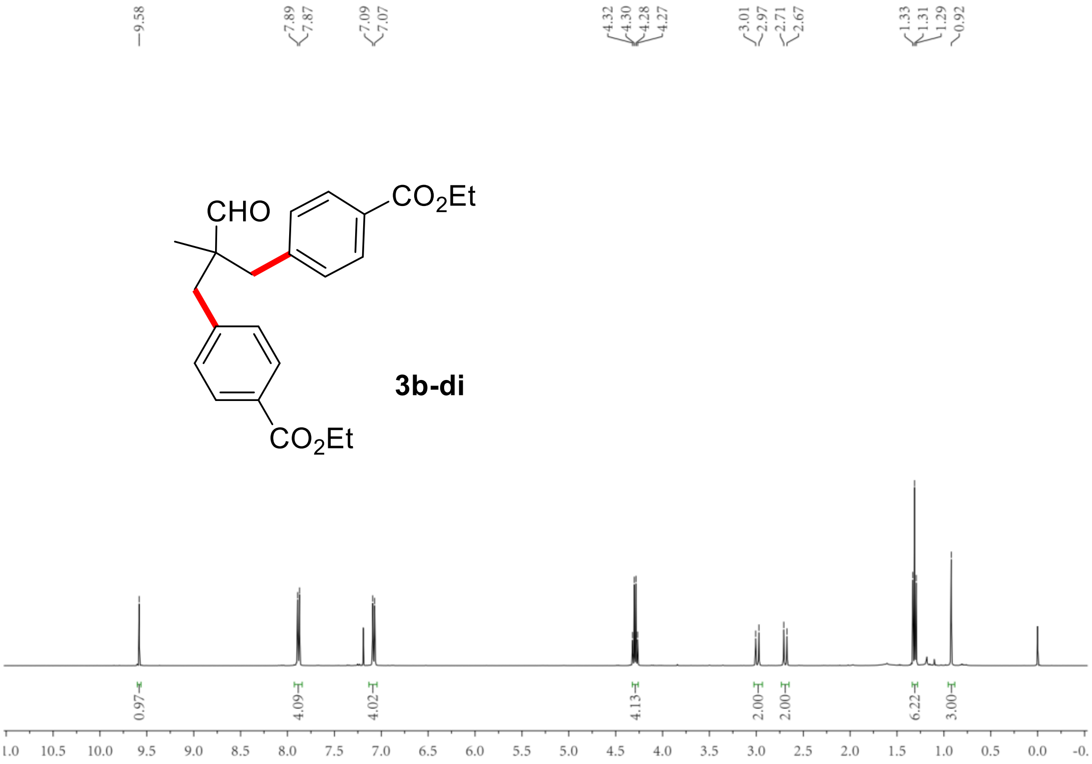

Figure S8 ^{1}H NMR spectrum (400MHz, $CDCl_3$, 298K) of **3b-di**

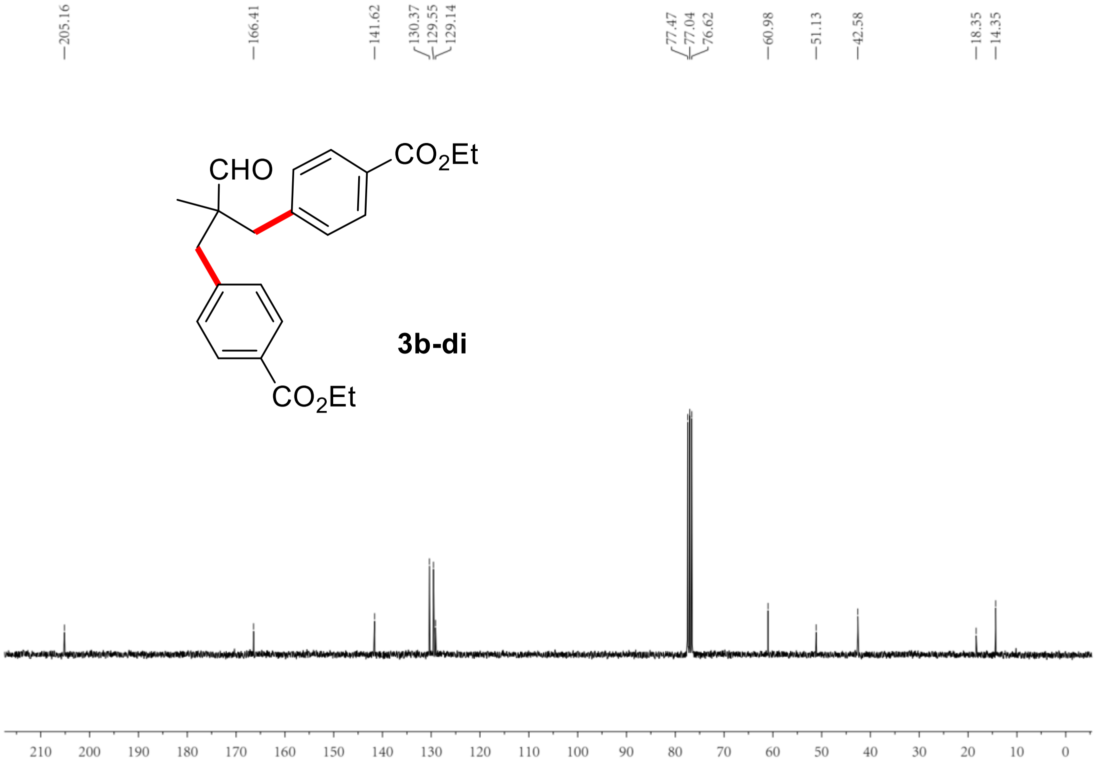

Figure S9 ^{13}C NMR spectrum (75MHz, $CDCl_3$, 298K) of 3**b-di**

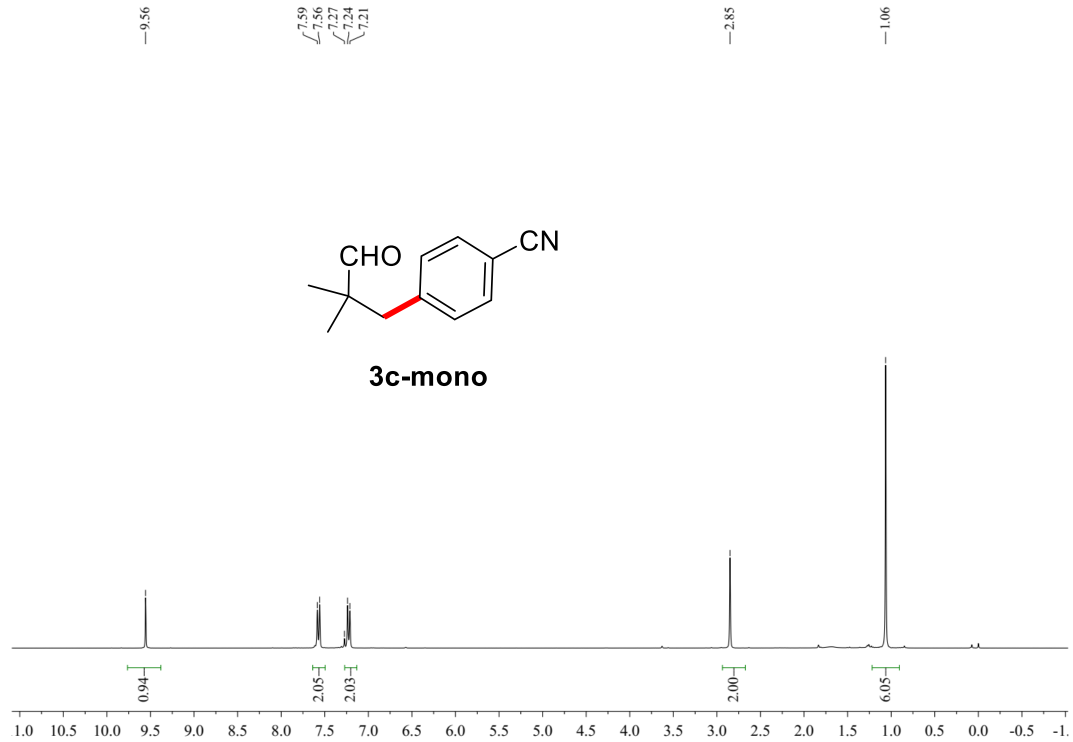

Figure S10 ^{1}H NMR spectrum (300MHz, $CDCl_3$, 298K) of **3c-mono**

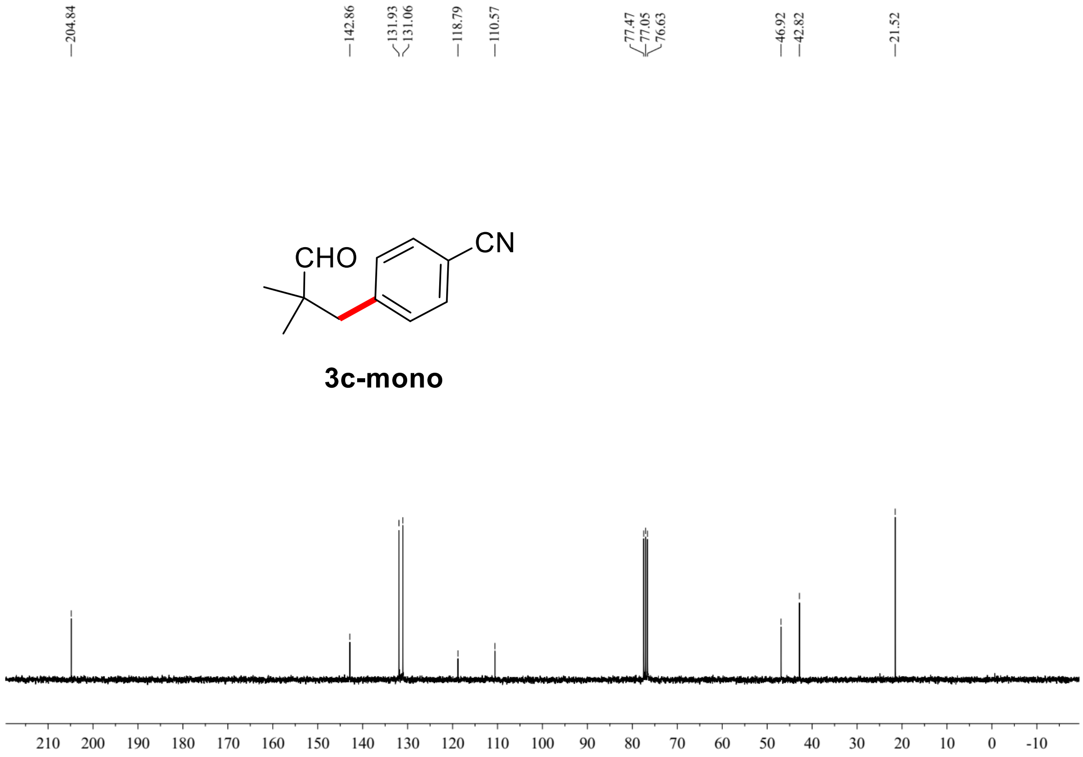

Figure S11 ^{13}C NMR spectrum (75MHz, $CDCl_3$, 298K) of **3c-mono**

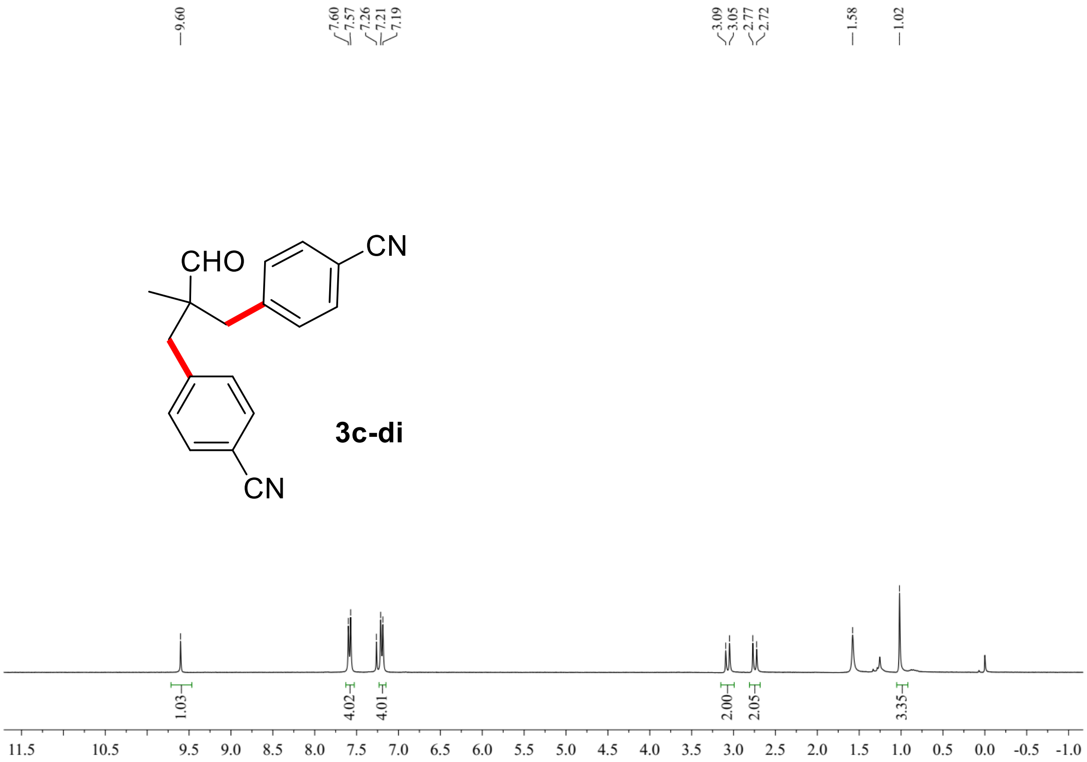

Figure S12 ^{1}H NMR spectrum (300MHz, $CDCl_3$, 298K) of **3c-di**

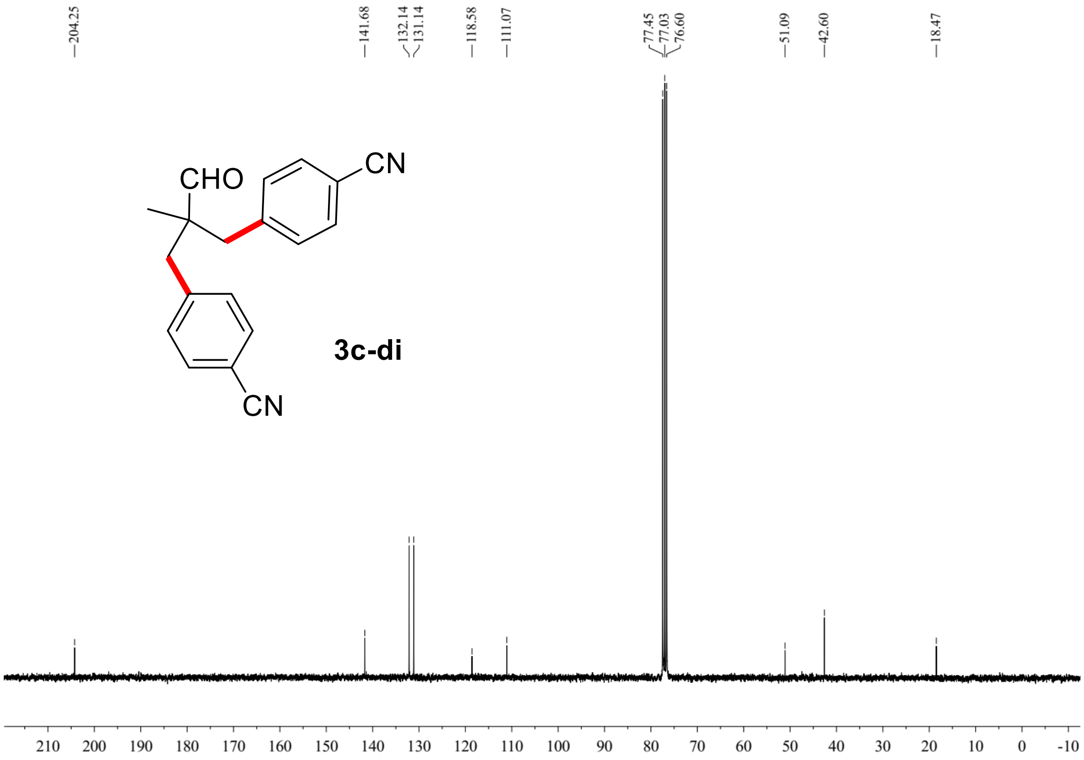

Figure S13 ^{13}C NMR spectrum (75MHz, $CDCl_3$, 298K) of 3**c-di**

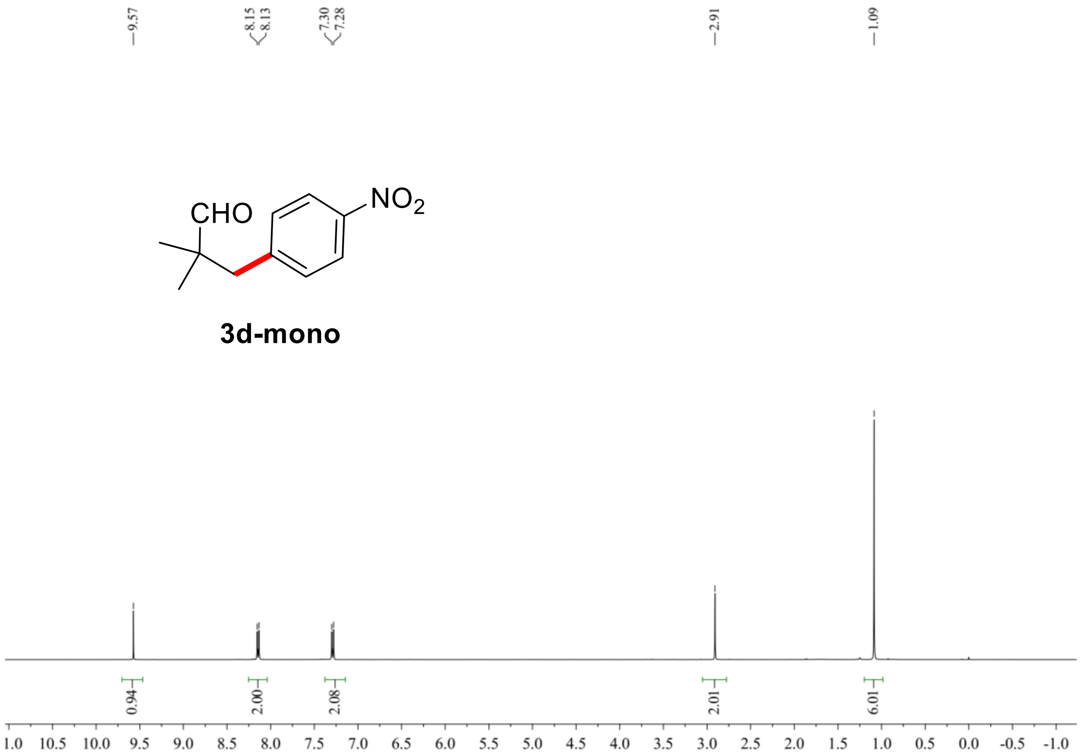

Figure S14 ^{1}H NMR spectrum (300MHz, $CDCl_3$, 298K) of **3d-mono**

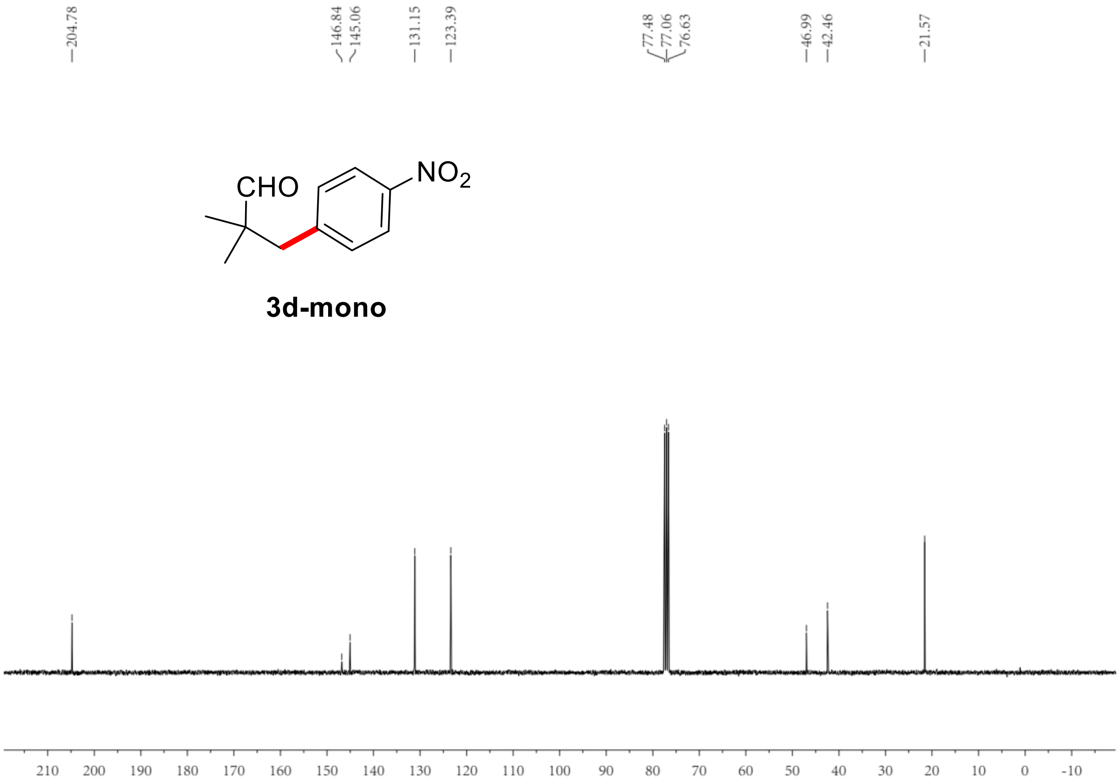

Figure S15 ^{13}C NMR spectrum (75MHz, $CDCl_3$, 298K) of **3d-mono**

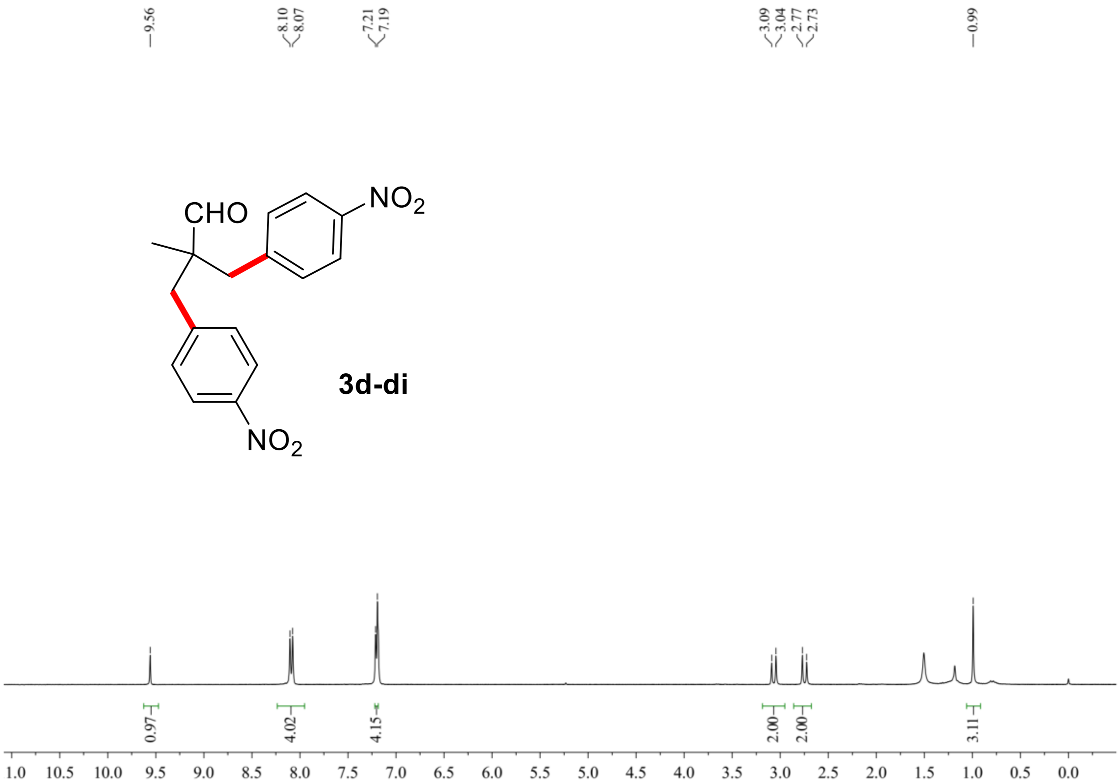

Figure S16 ^{1}H NMR spectrum (300MHz, CDCl3, 298K) of **3d-di**

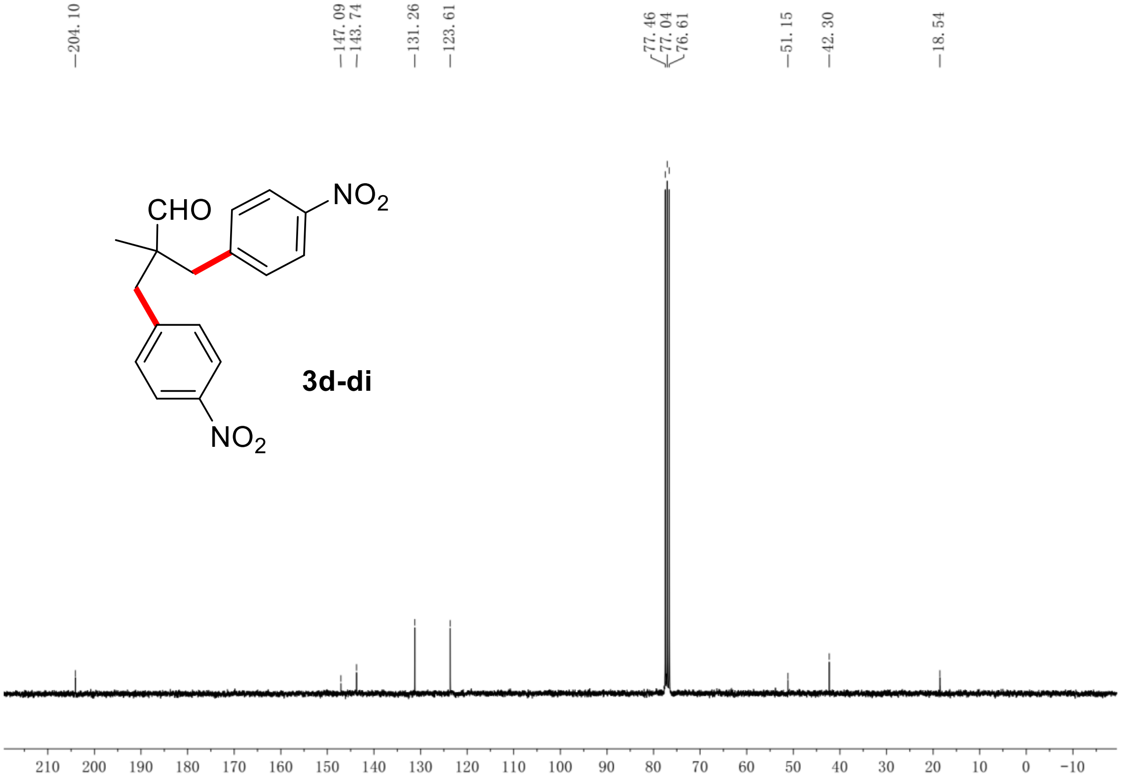

Figure S17 ^{13}C NMR spectrum (75MHz, CDCl3, 298K) of **3d-di**

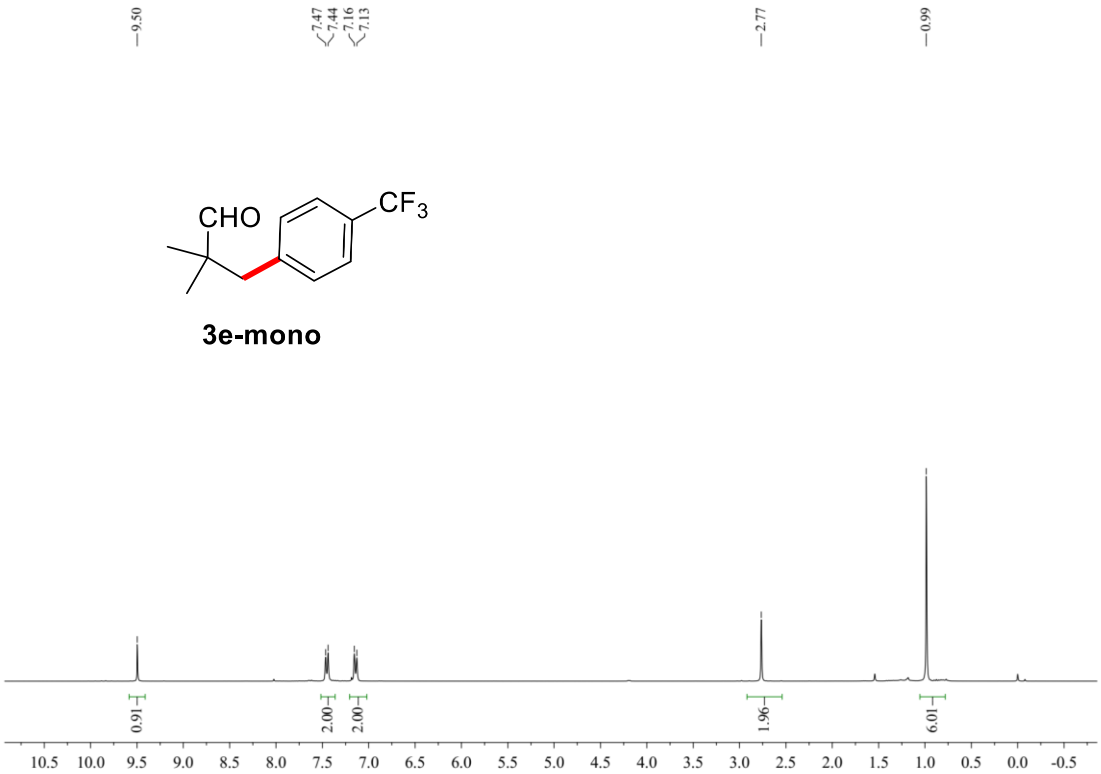

Figure S18 ^{1}H NMR spectrum (300MHz, $CDCl_3$, 298K) of **3e-mono**

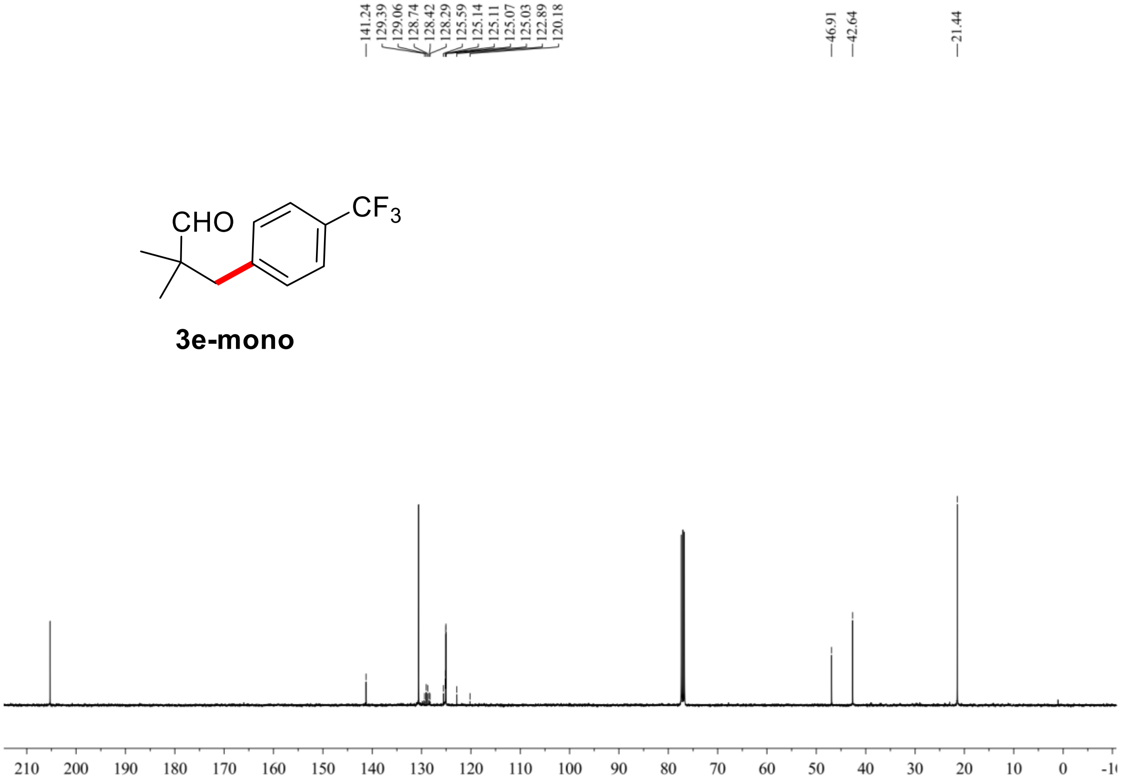

Figure S19 ^{13}C NMR spectrum (101MHz, $CDCl_3$, 298K) of **3e-mono**

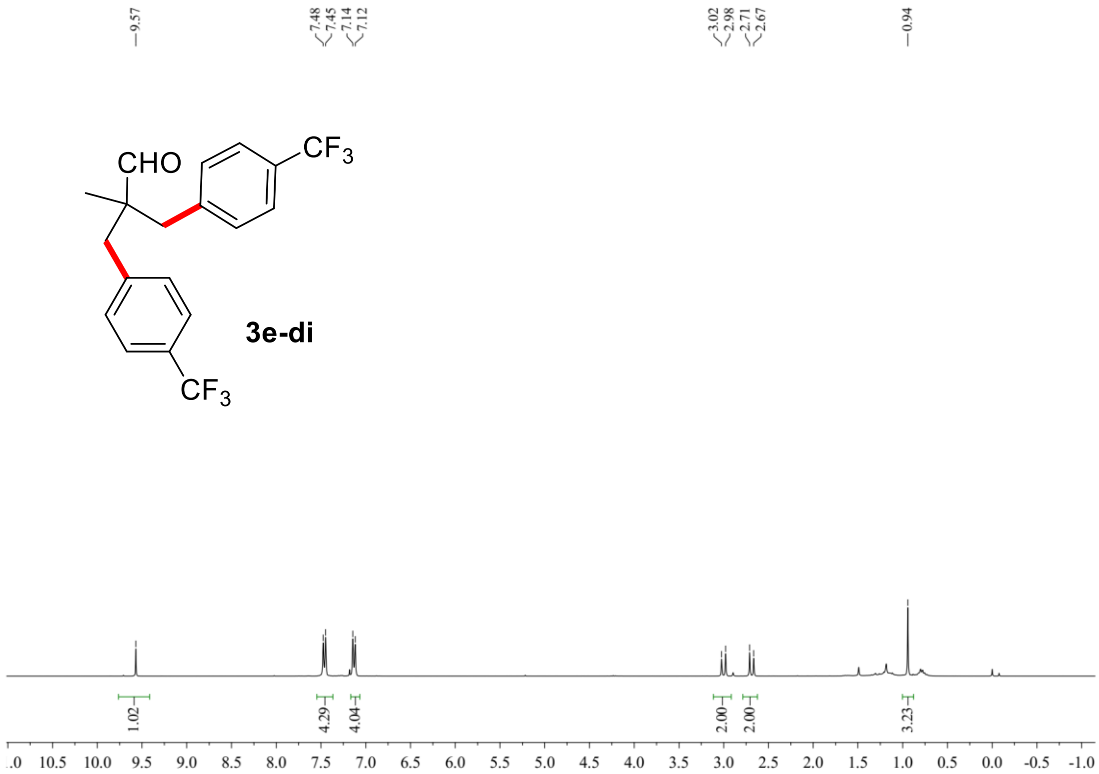

Figure S20 ^{1}H NMR spectrum (300MHz, $CDCl_3$, 298K) of **3e-di**

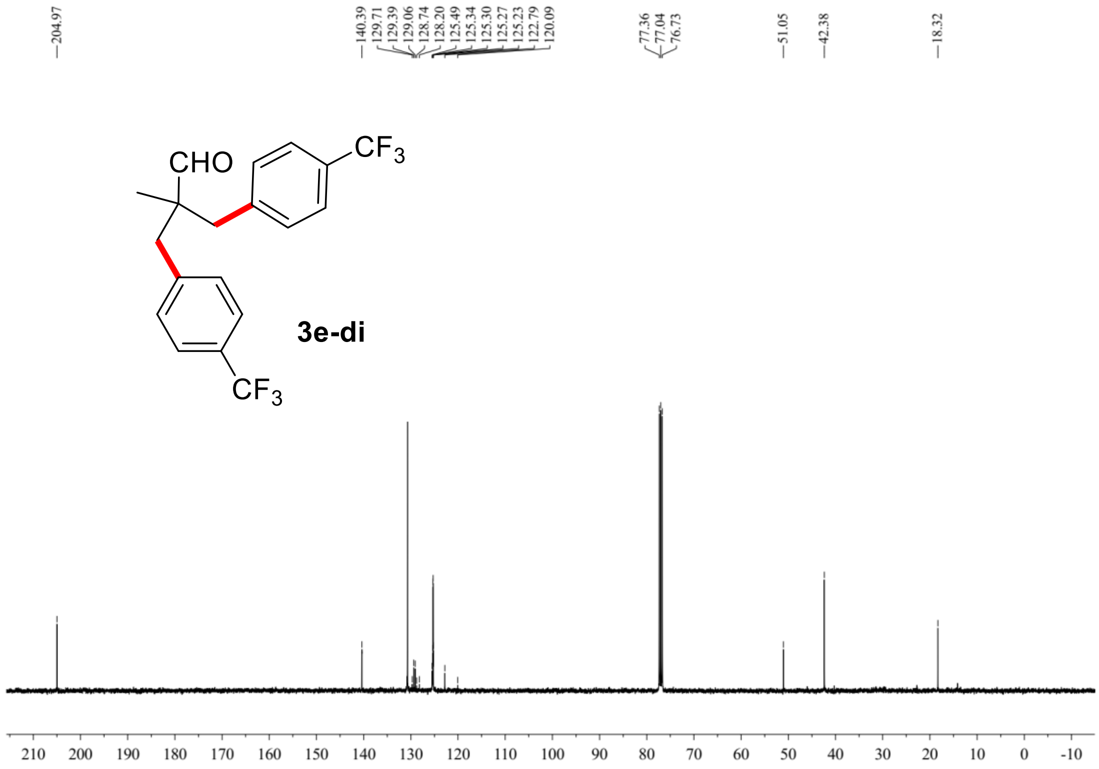

Figure S21 ^{13}C NMR spectrum (101MHz, $CDCl_3$, 298K) of **3e-di**

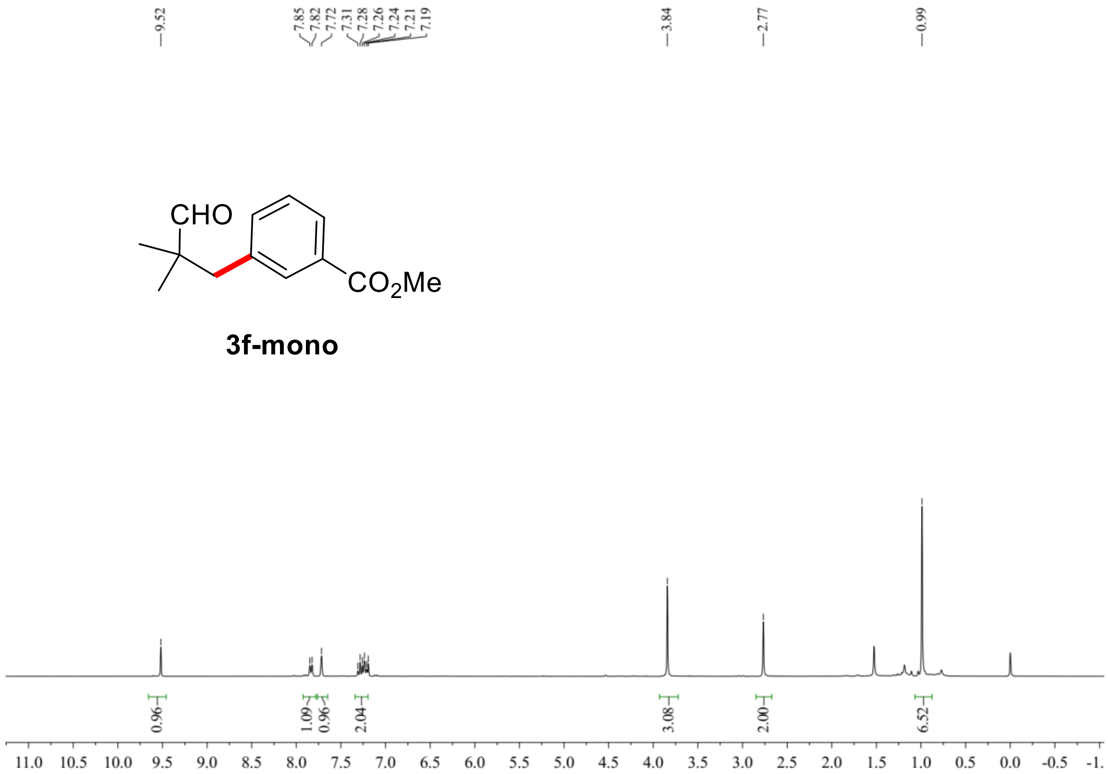

Figure S22 ^{1}H NMR spectrum (300MHz, $CDCl_3$, 298K) of **3f-mono**

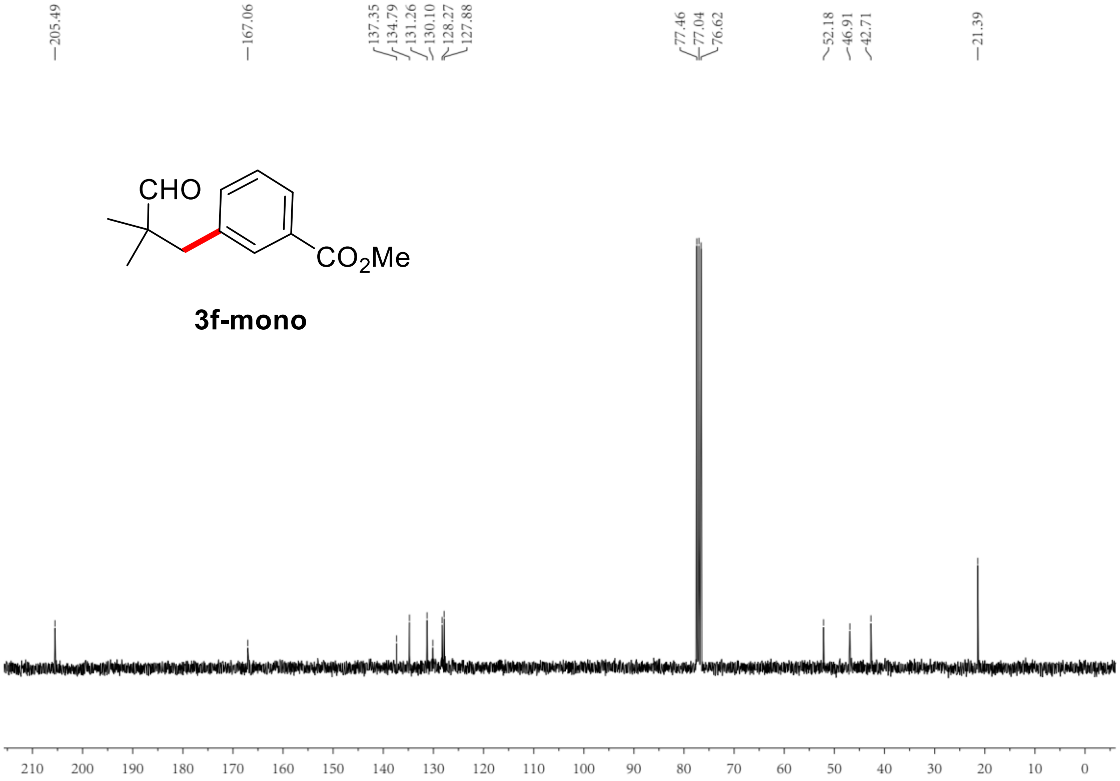

Figure S23 ^{13}C NMR spectrum (75MHz, $CDCl_3$, 298K) of **3f-mono**

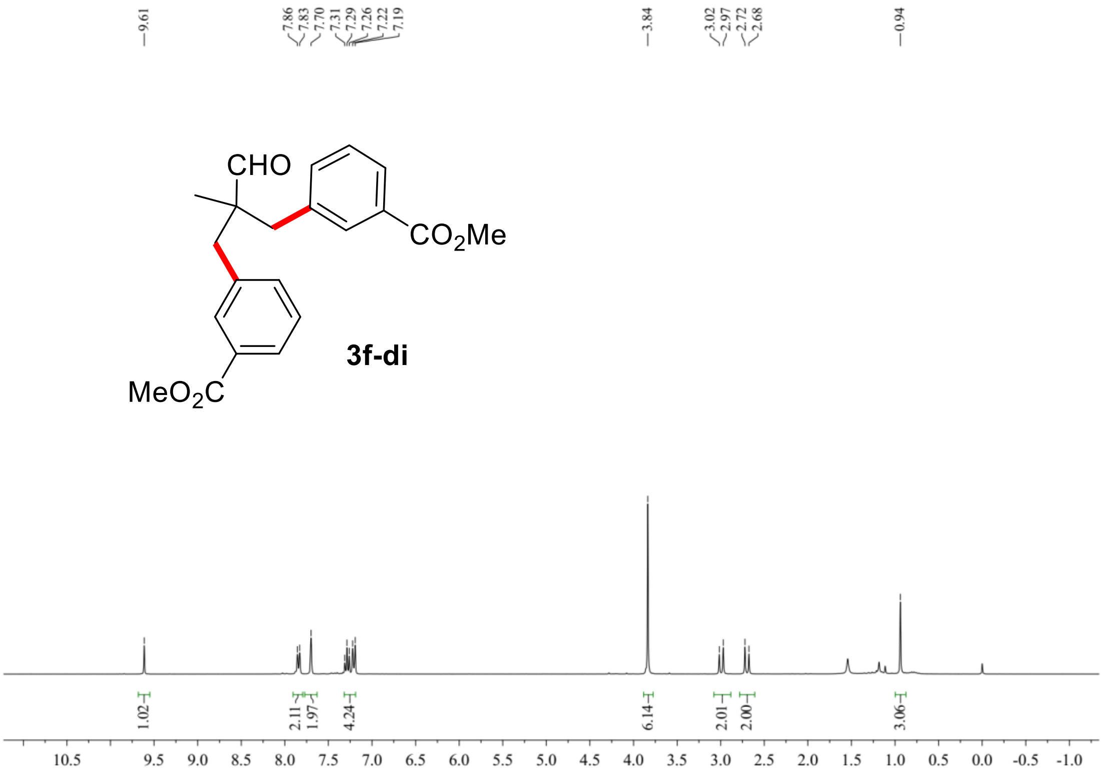

Figure S24 ^{1}H NMR spectrum (300MHz, $CDCl_3$, 298K) of **3f-di**

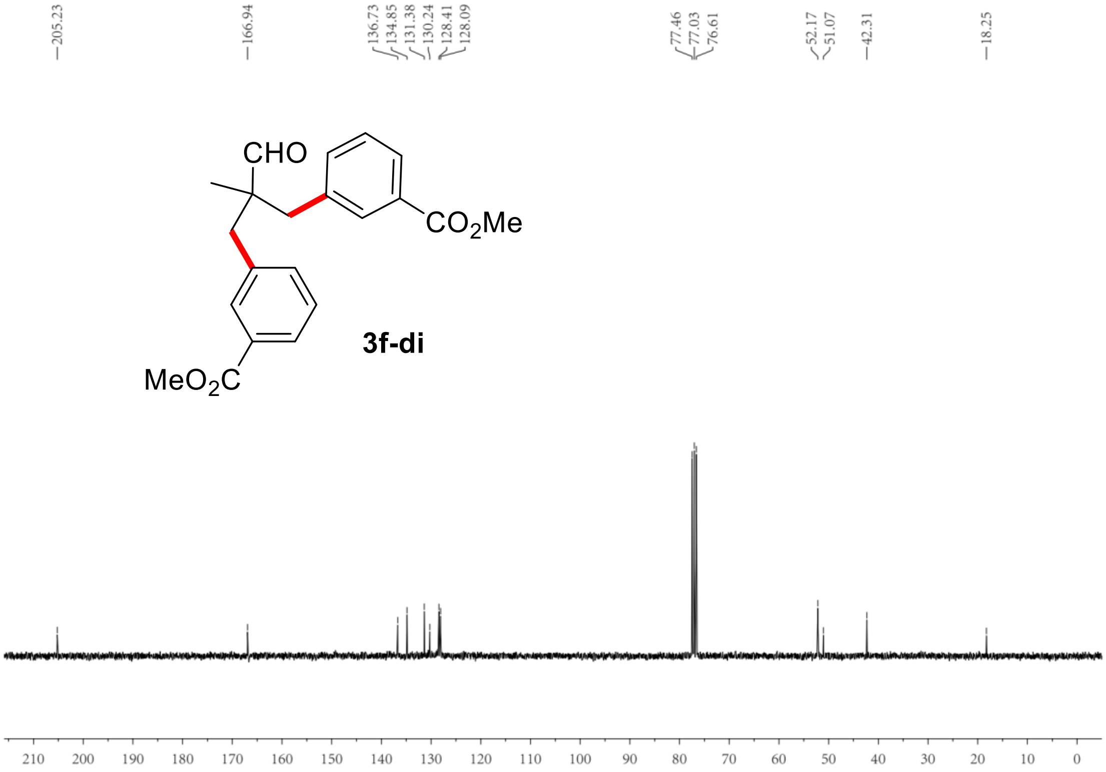

Figure S25 ^{13}C NMR spectrum (75MHz, $CDCl_3$, 298K) of **3f-di**

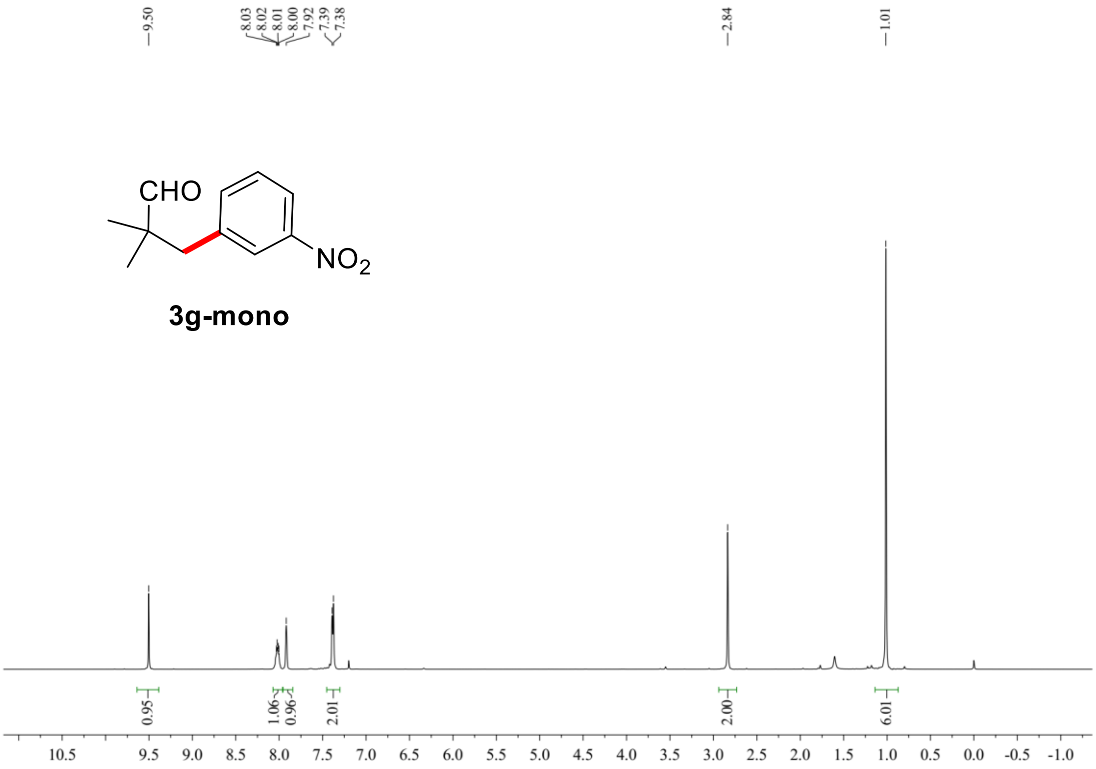

Figure S26 ^{1}H NMR spectrum (300MHz, $CDCl_3$, 298K) of **3g-mono**

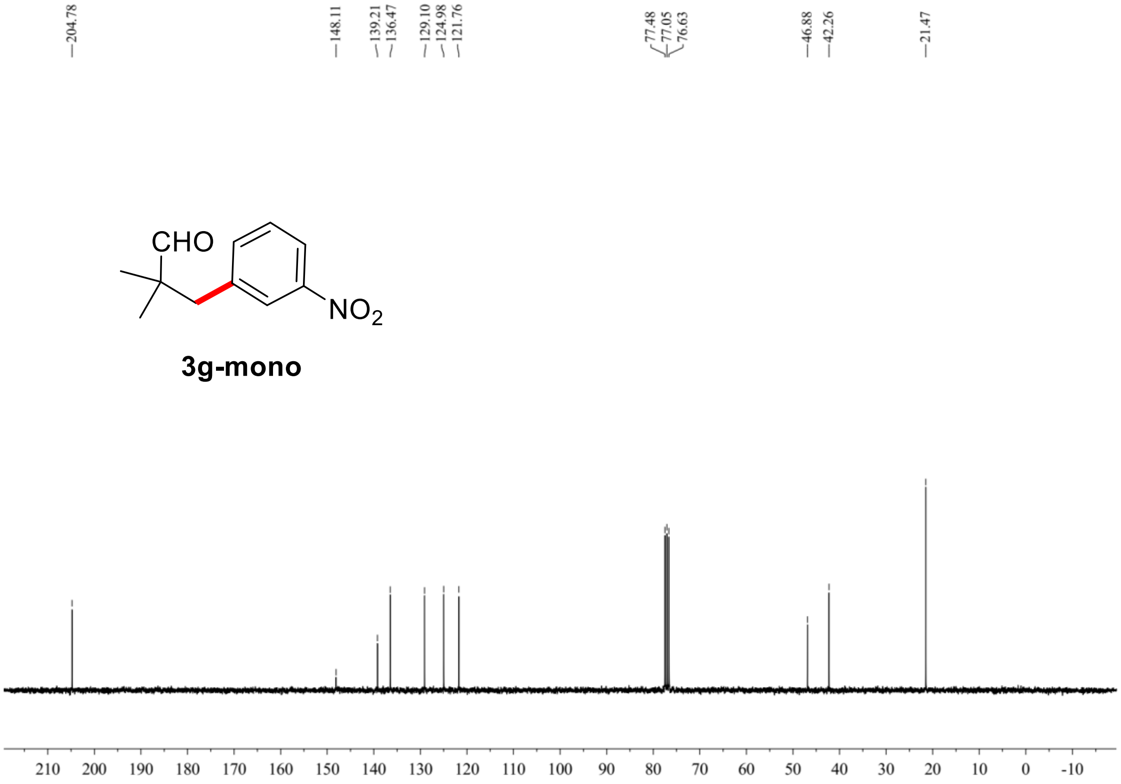

Figure S27 ^{13}C NMR spectrum (75MHz, $CDCl_3$, 298K) of **3g-mono**

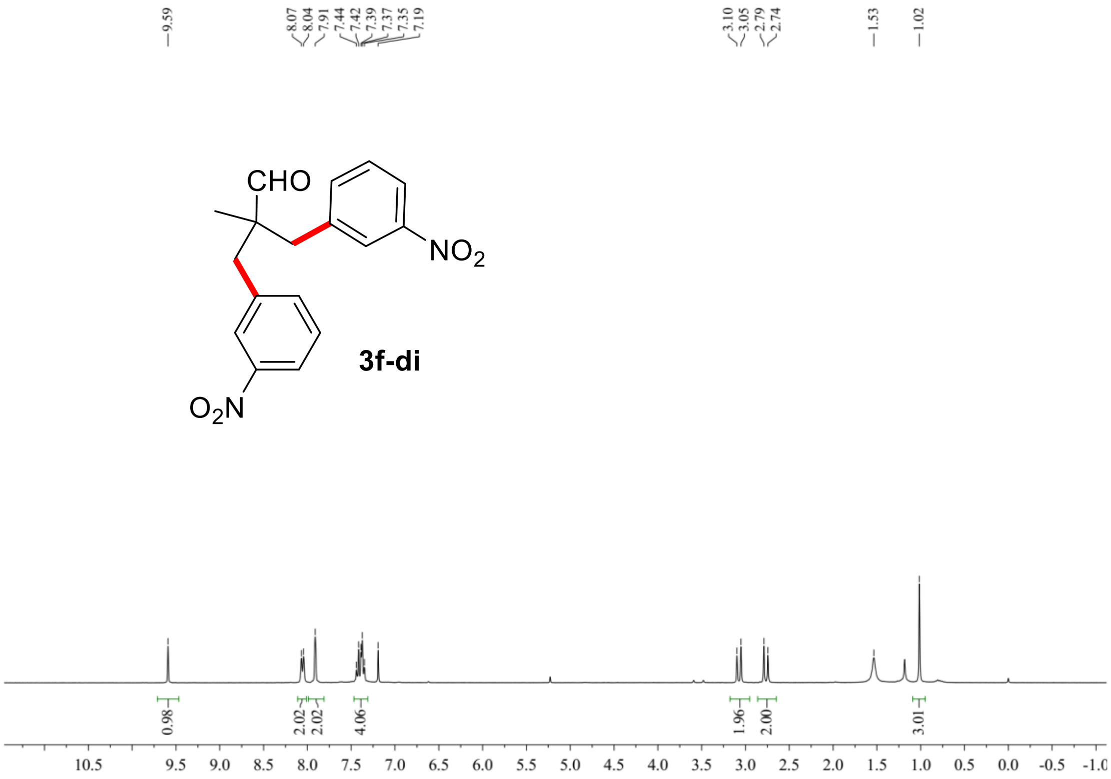

Figure S28 ^{1}H NMR spectrum (300MHz, $CDCl_3$, 298K) of **3g-di**

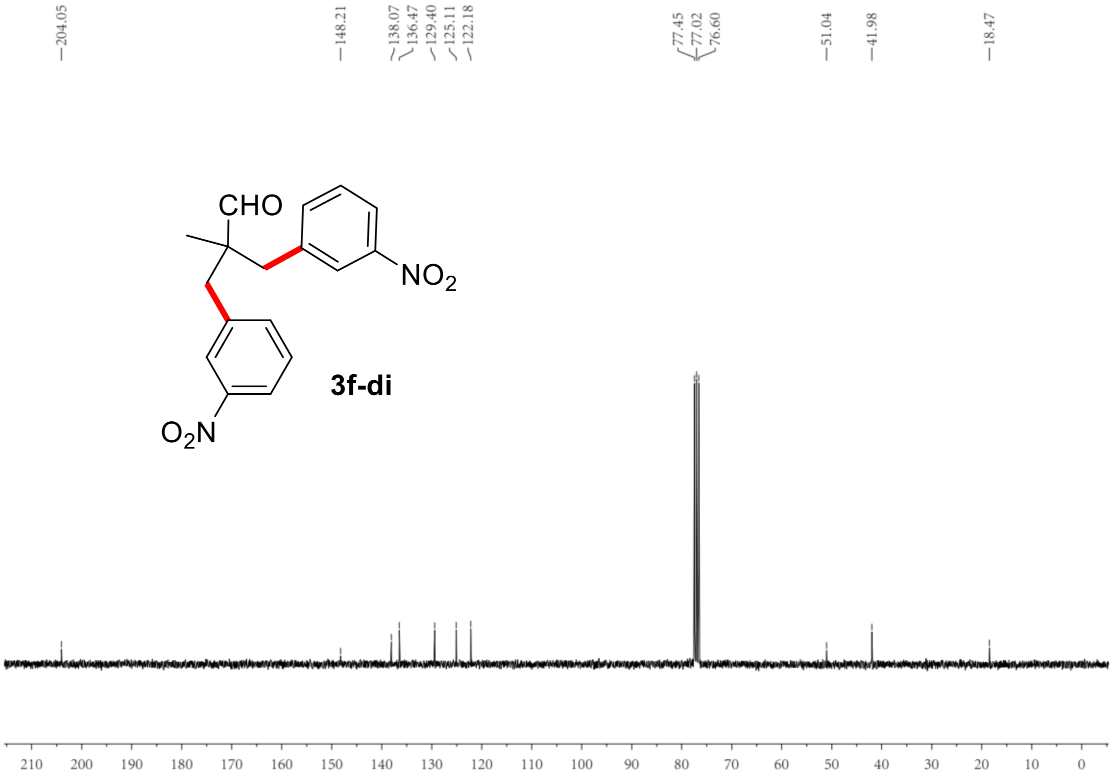

Figure S29 ^{13}C NMR spectrum (75MHz, $CDCl_3$, 298K) of **3g-di**

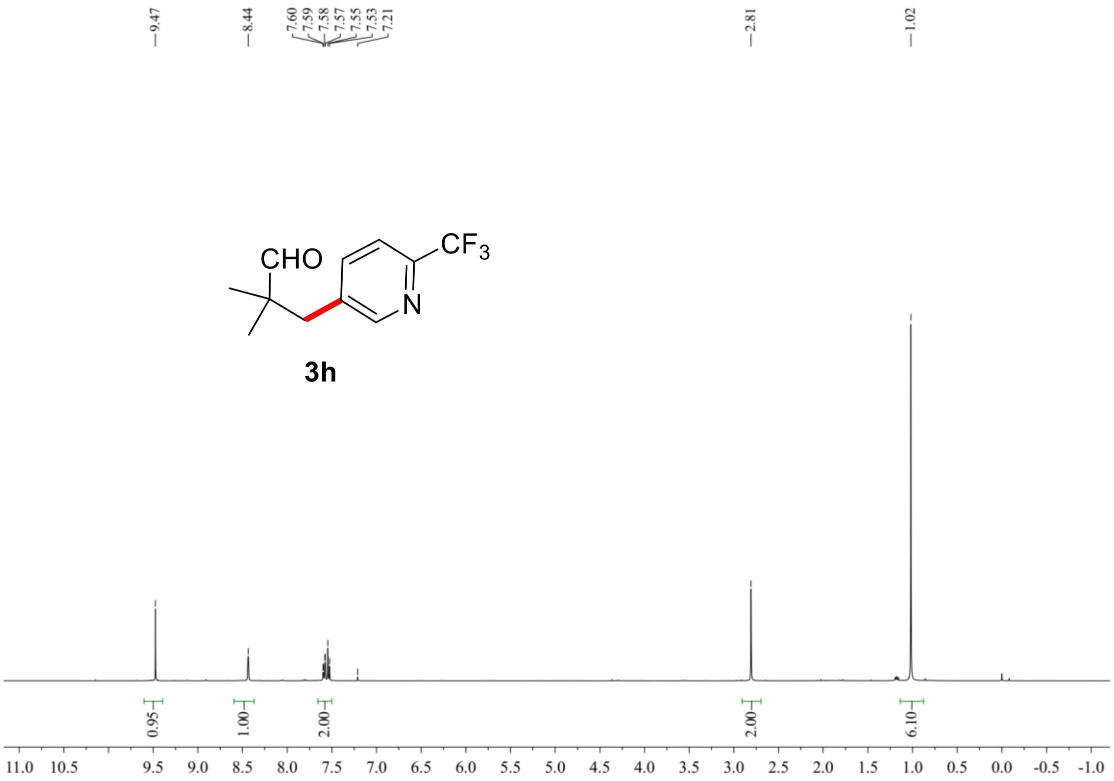

Figure S30 ^{1}H NMR spectrum (400MHz, $CDCl_3$, 298K) of **3h**

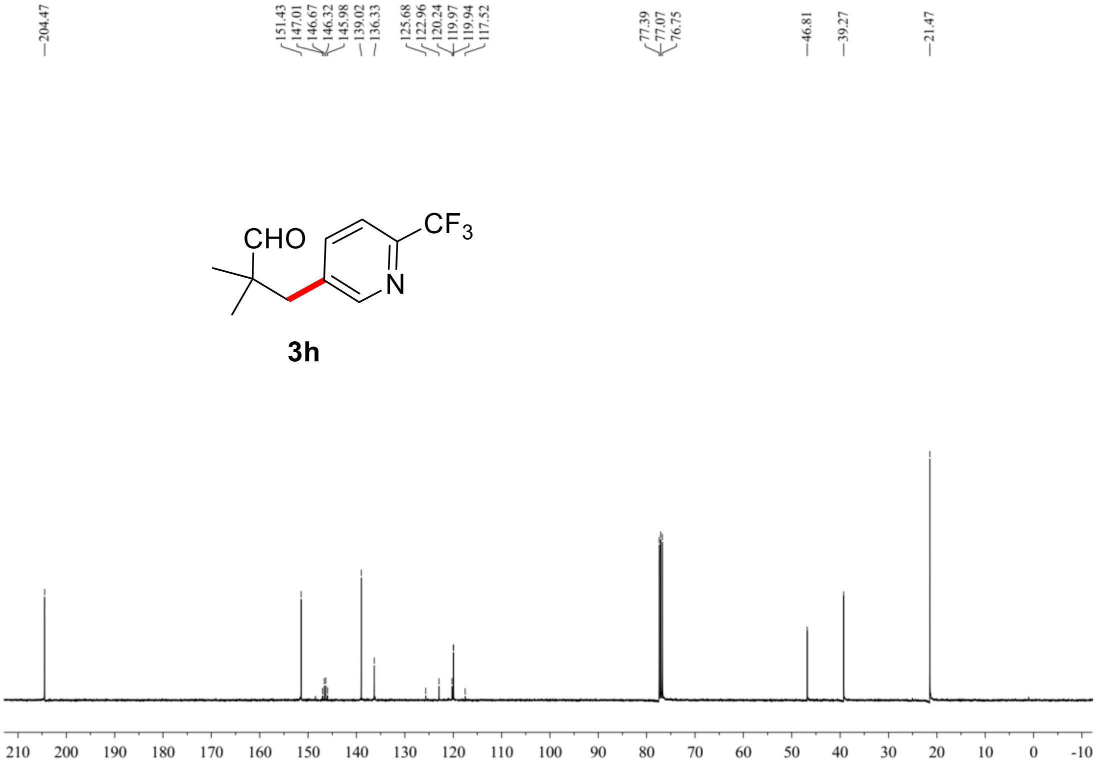

Figure S31 ^{13}C NMR spectrum (101MHz, $CDCl_3$, 298K) of **3h**

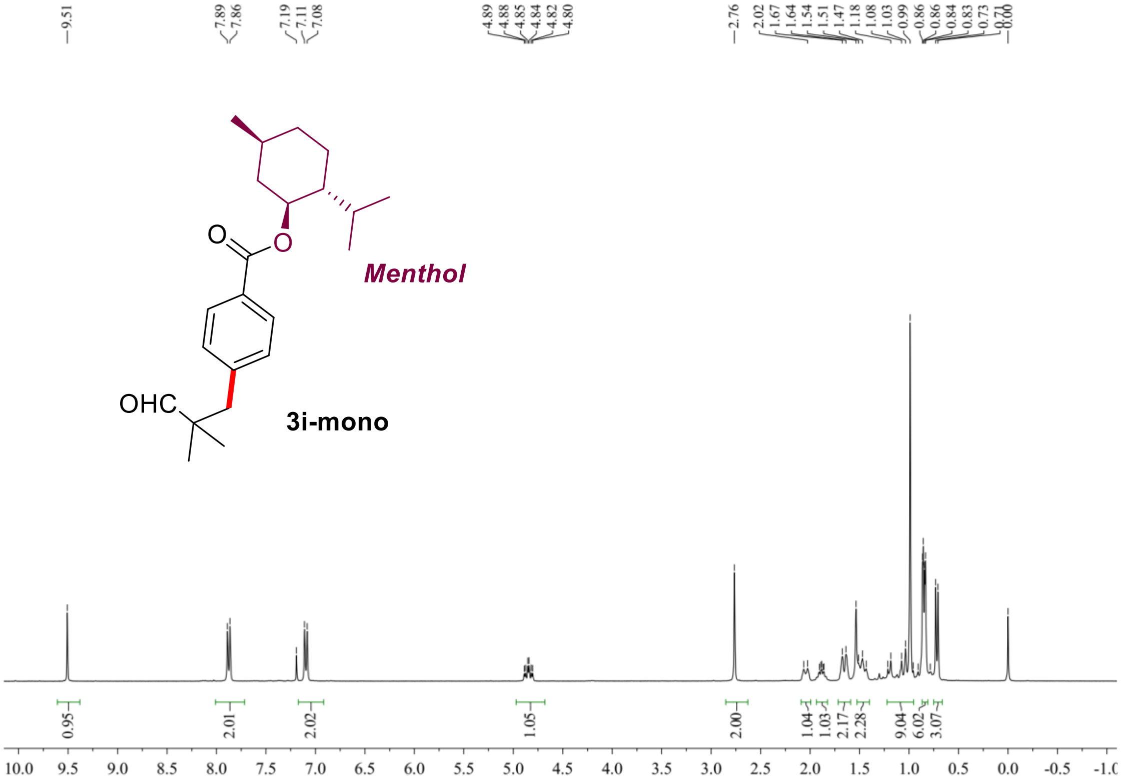

Figure S32 ^{1}H NMR spectrum (300MHz, $CDCl_3$, 298K) of **3i-mono**

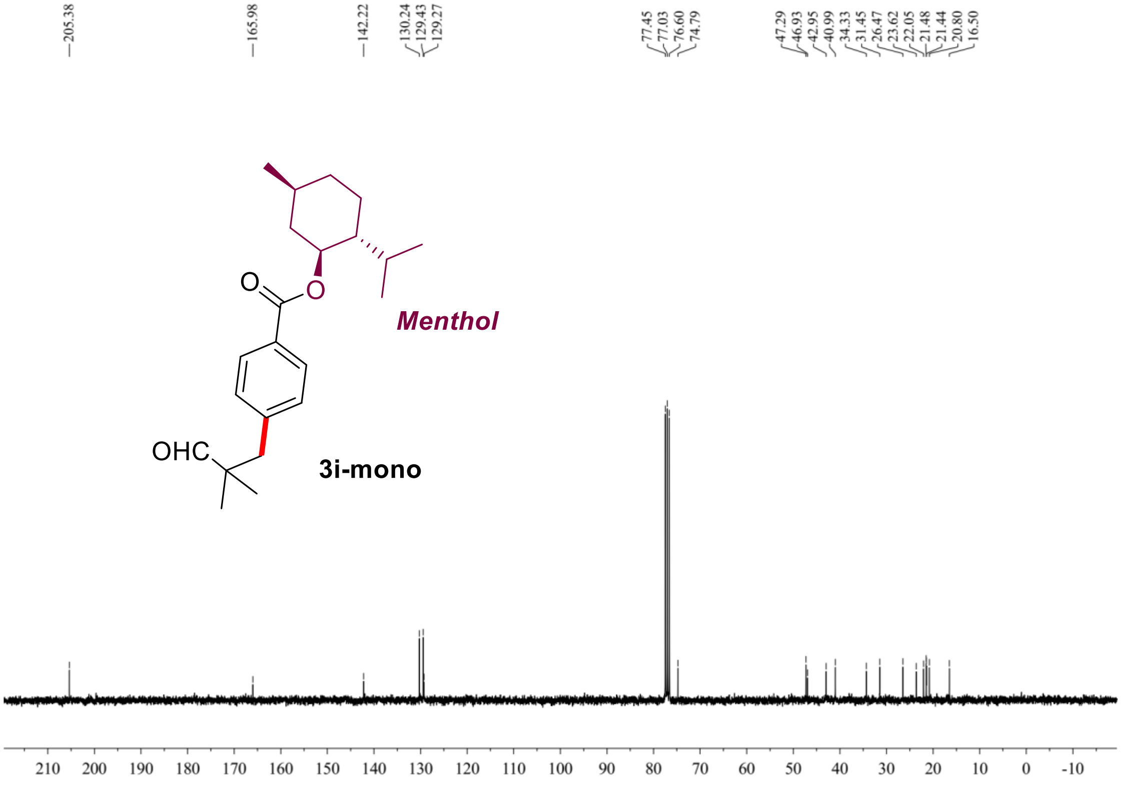

Figure S33 ^{13}C NMR spectrum (75MHz, $CDCl_3$, 298K) of **3i-mono**

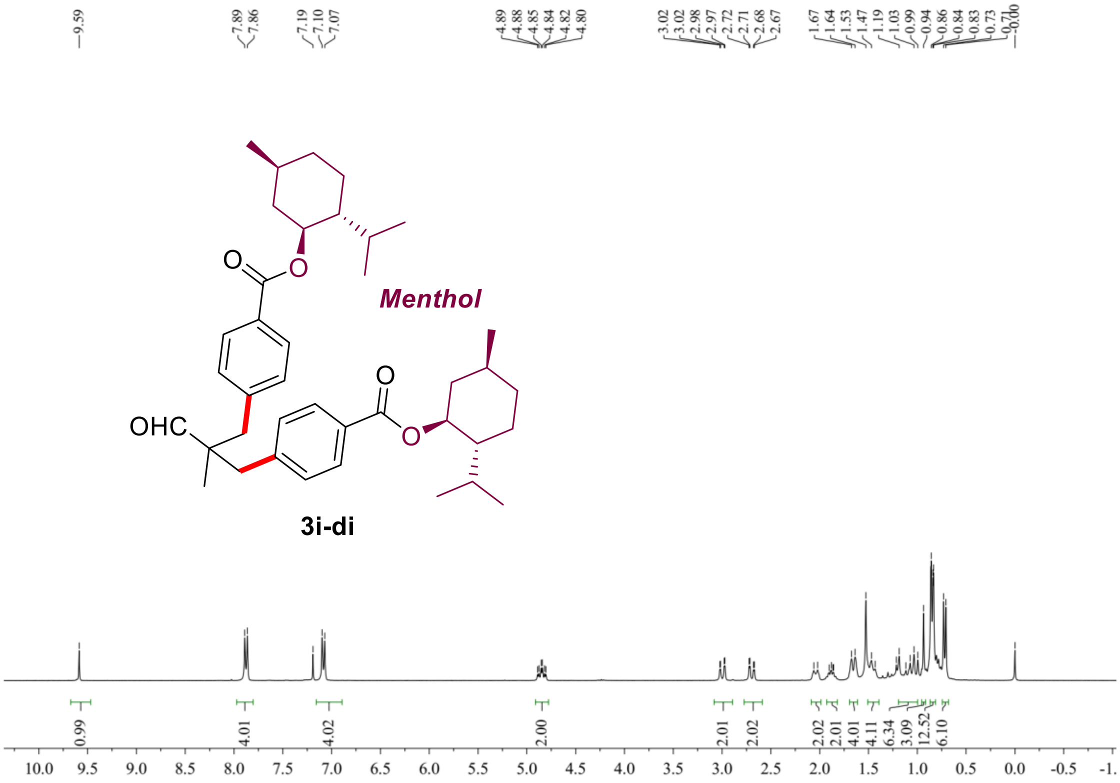

Figure S34 ^{1}H NMR spectrum (300MHz, $CDCl_3$, 298K) of **3i-di**

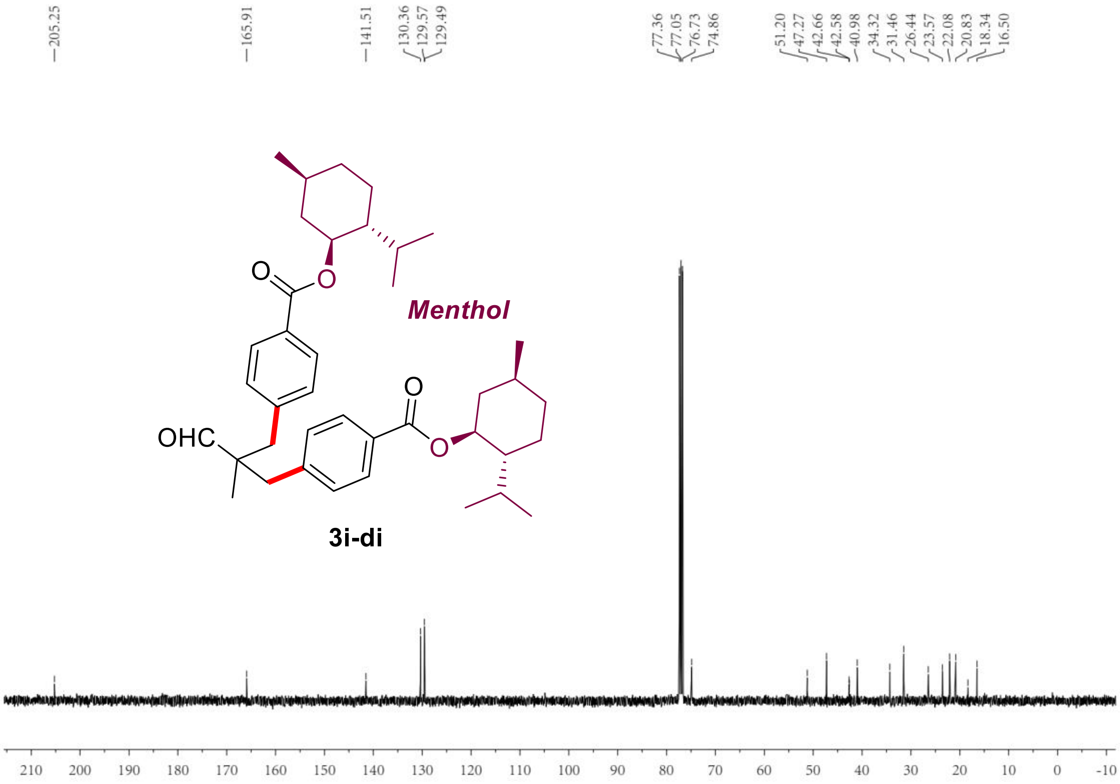

Figure S35 ^{13}C NMR spectrum (101MHz, $CDCl_3$, 298K) of **3i-di**

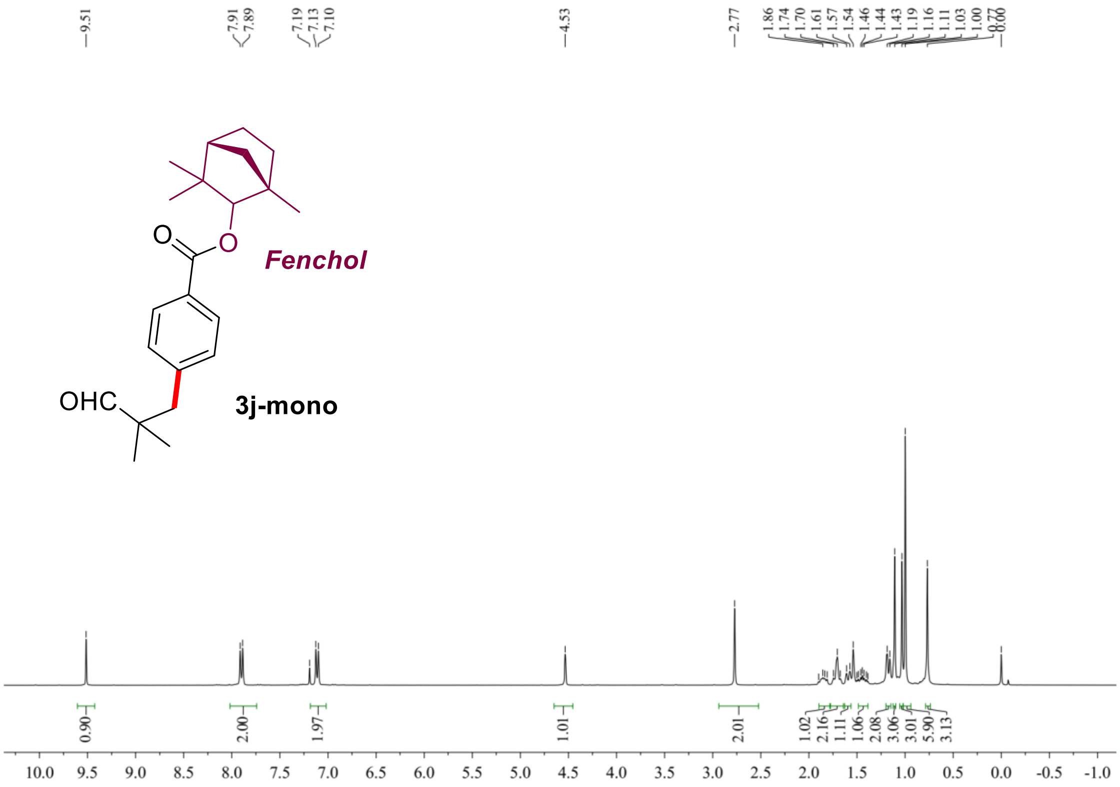

Figure S36 ^{1}H NMR spectrum (300MHz, $CDCl_3$, 298K) of **3j-mono**

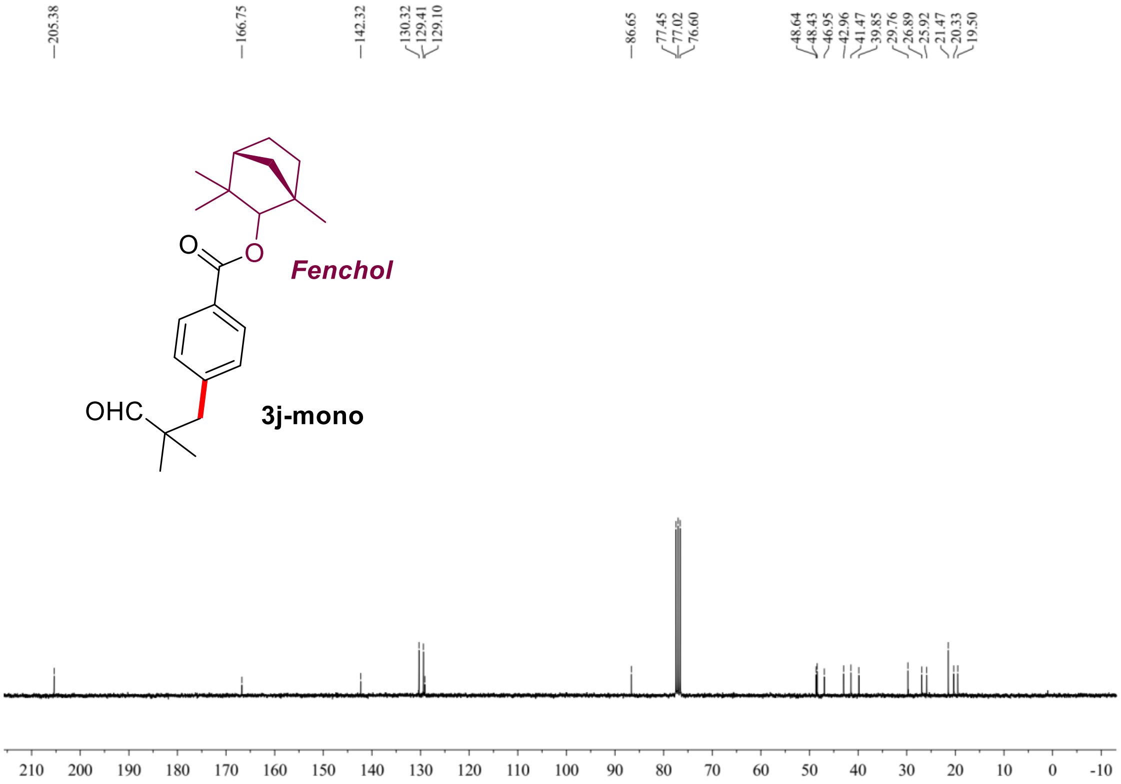

Figure S37 ^{13}C NMR spectrum (75MHz, $CDCl_3$, 298K) of **3j-mono**

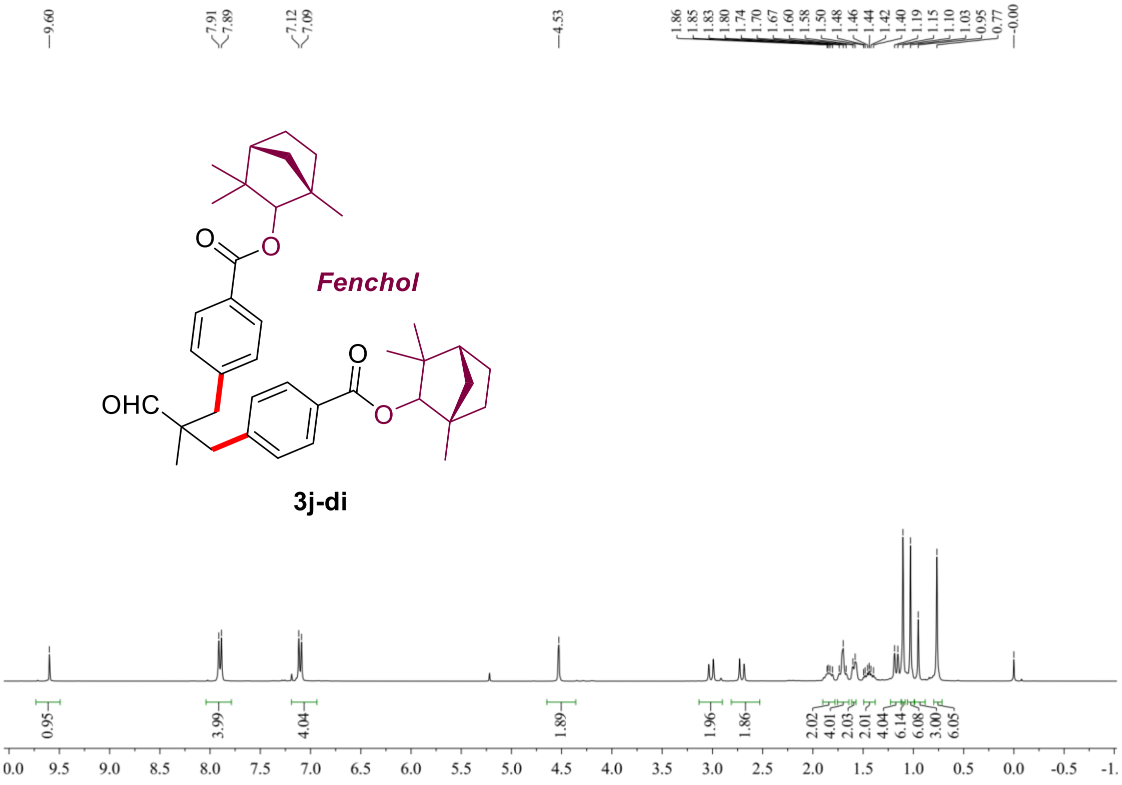

Figure S38 ¹H NMR spectrum (300MHz, CDCl₃, 298K) of **3j-di**

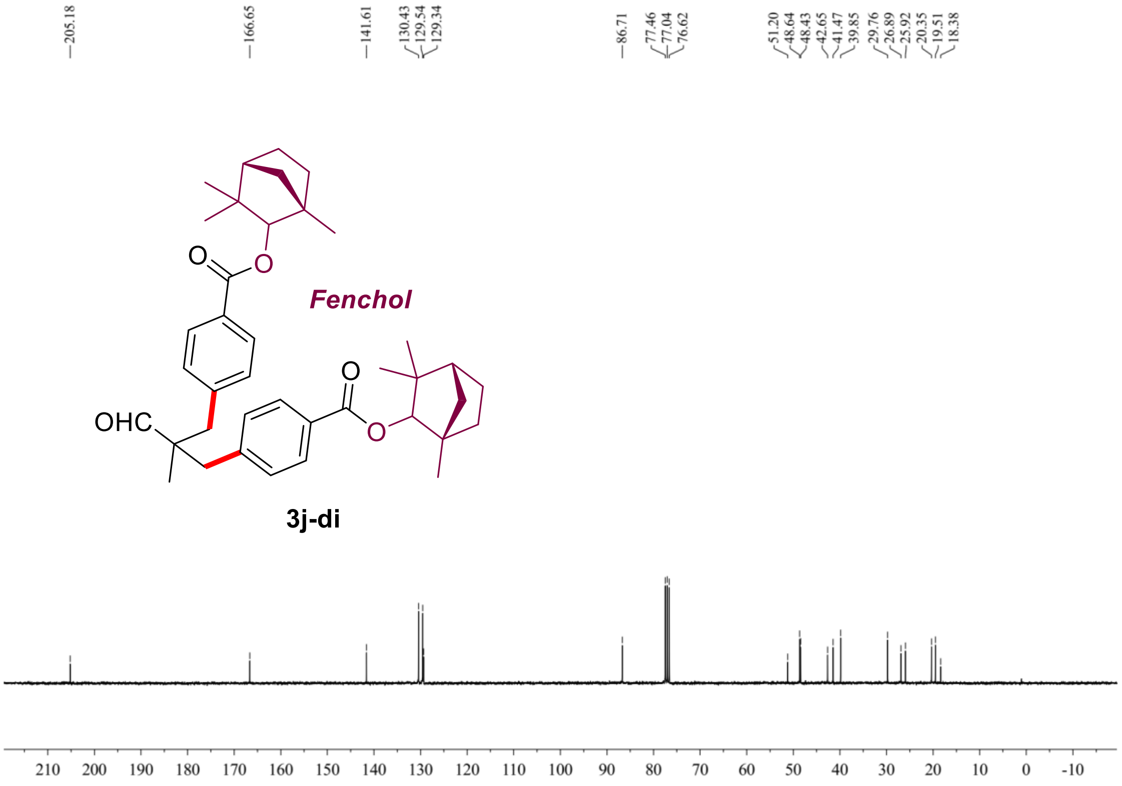

Figure S39 ¹³C NMR spectrum (75MHz, CDCl₃, 298K) of **3j-di**

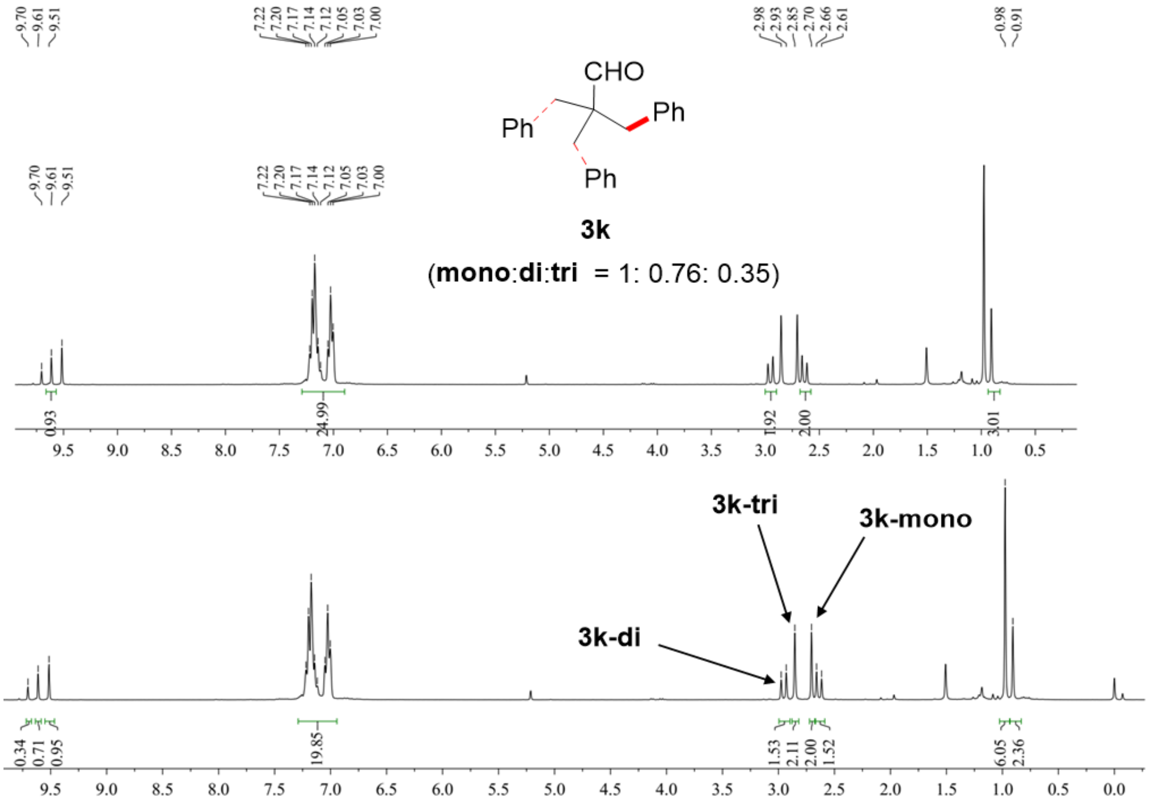

Figure S40 ^{1}H NMR spectrum (300MHz, $CDCl_3$, 298K) of **3k**

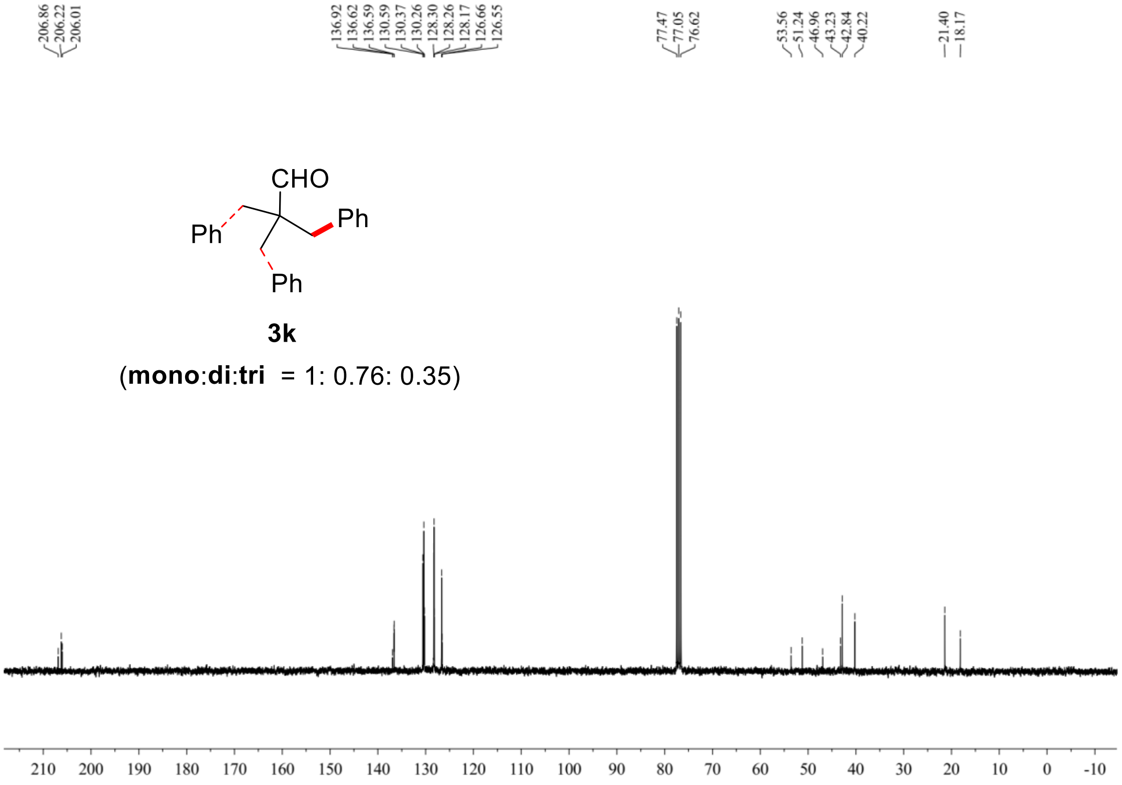

Figure S41 ^{13}C NMR spectrum (75MHz, $CDCl_3$, 298K) of **3k**

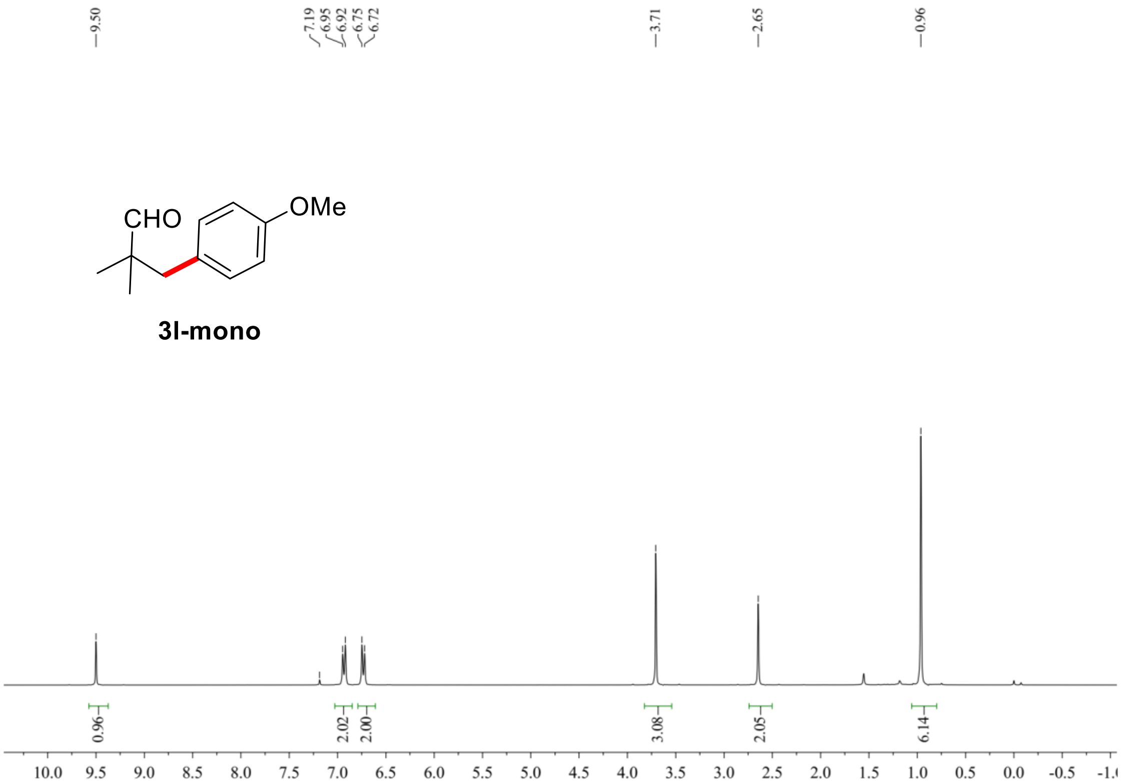

Figure S42 ^{1}H NMR spectrum (300MHz, $CDCl_3$, 298K) of **3l-mono**

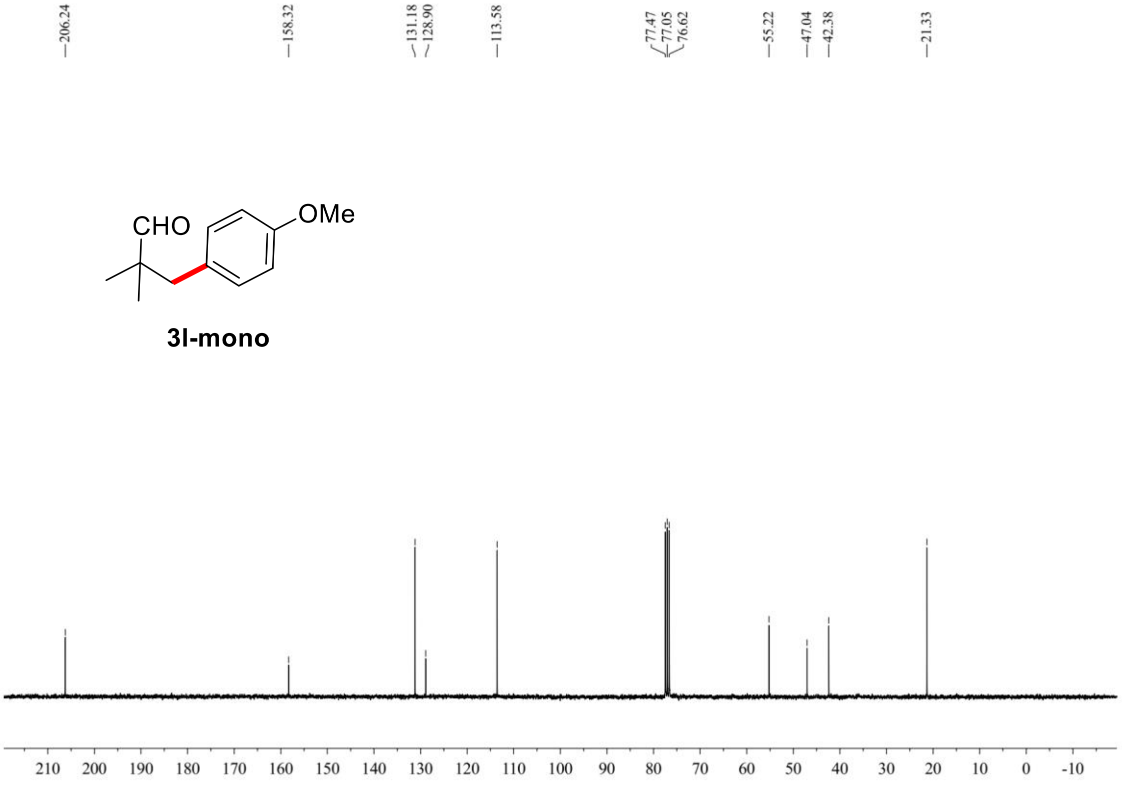

Figure S43 ^{13}C NMR spectrum (75MHz, $CDCl_3$, 298K) of **3l-mono**

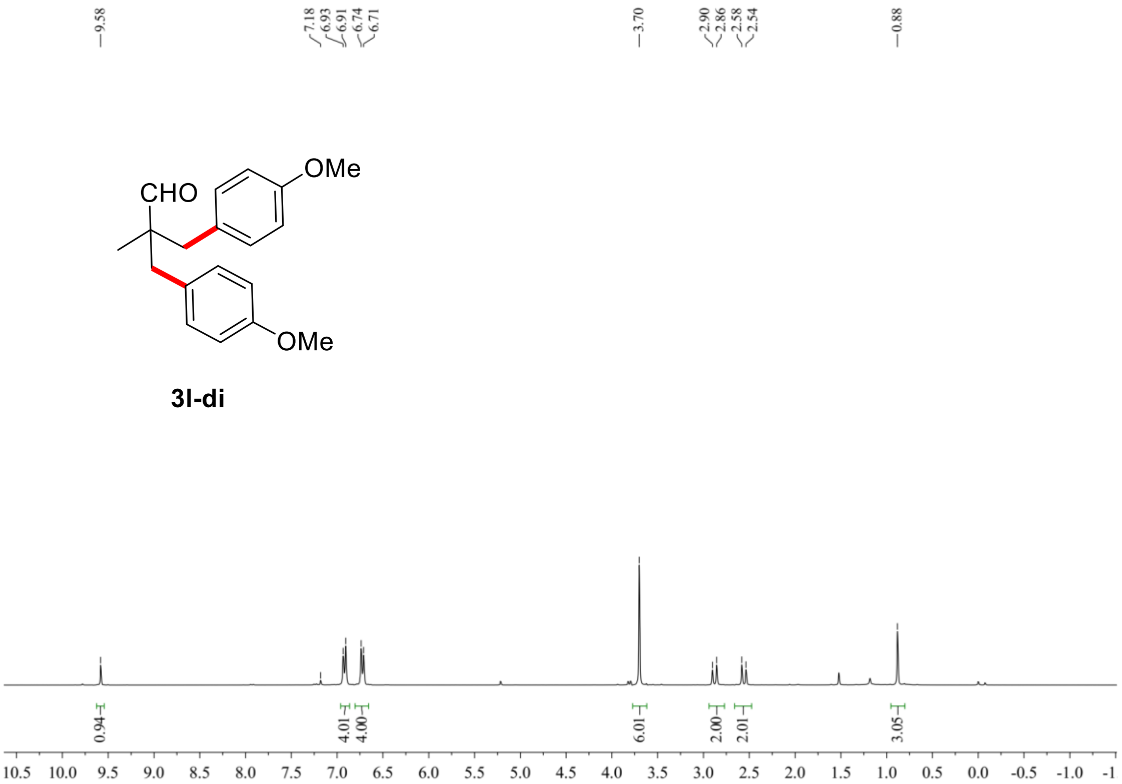

Figure S44 ^{1}H NMR spectrum (300MHz, $CDCl_3$, 298K) of **3l-di**

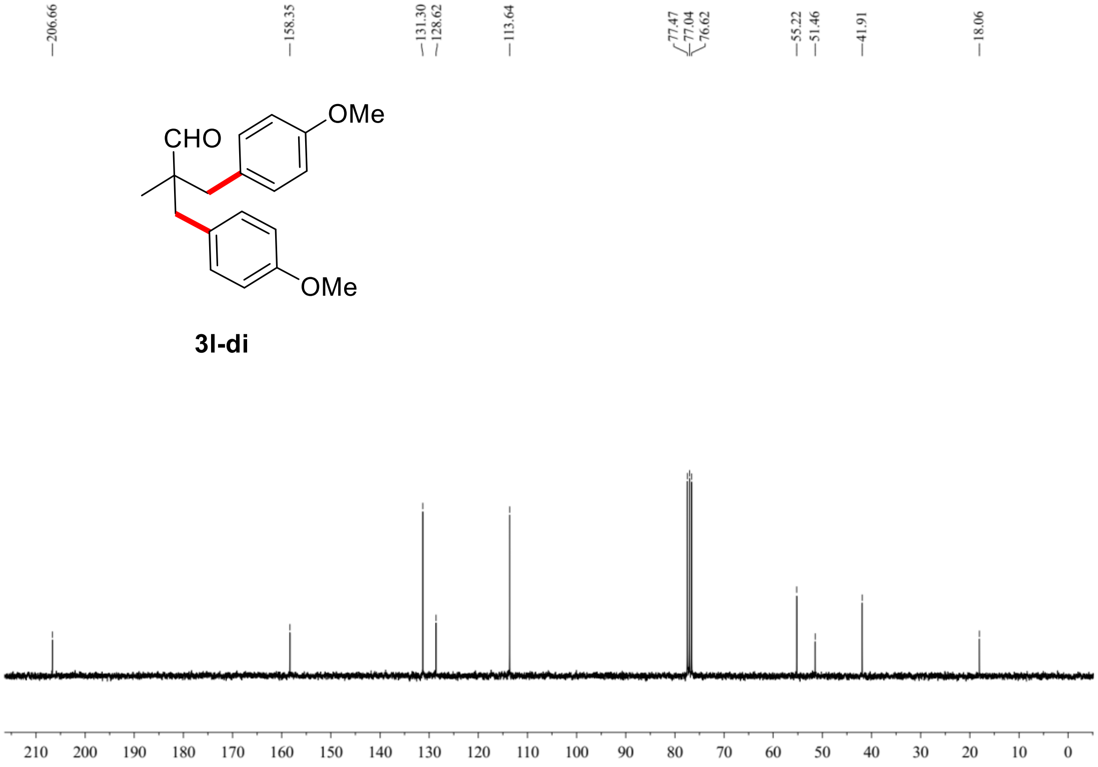

Figure S45 ^{13}C NMR spectrum (75MHz, $CDCl_3$, 298K) of **3l-di**

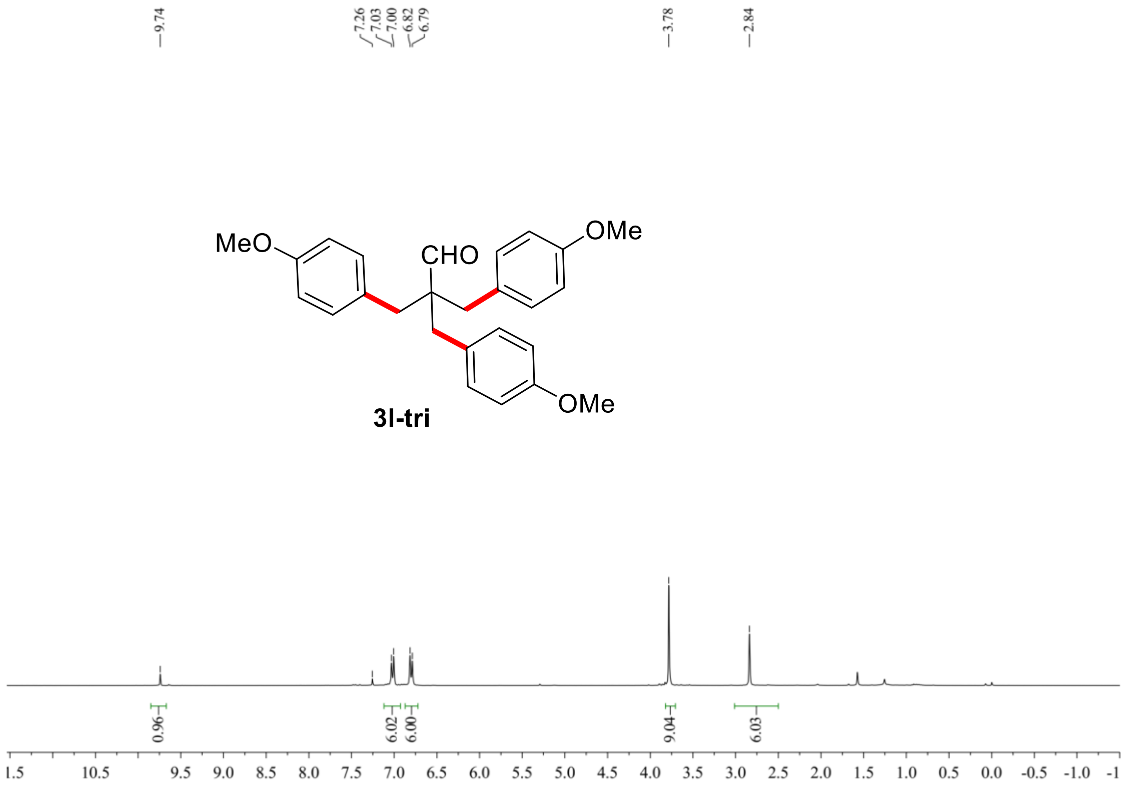

Figure S46 ^{1}H NMR spectrum (300MHz, $CDCl_3$, 298K) of **3l-tri**

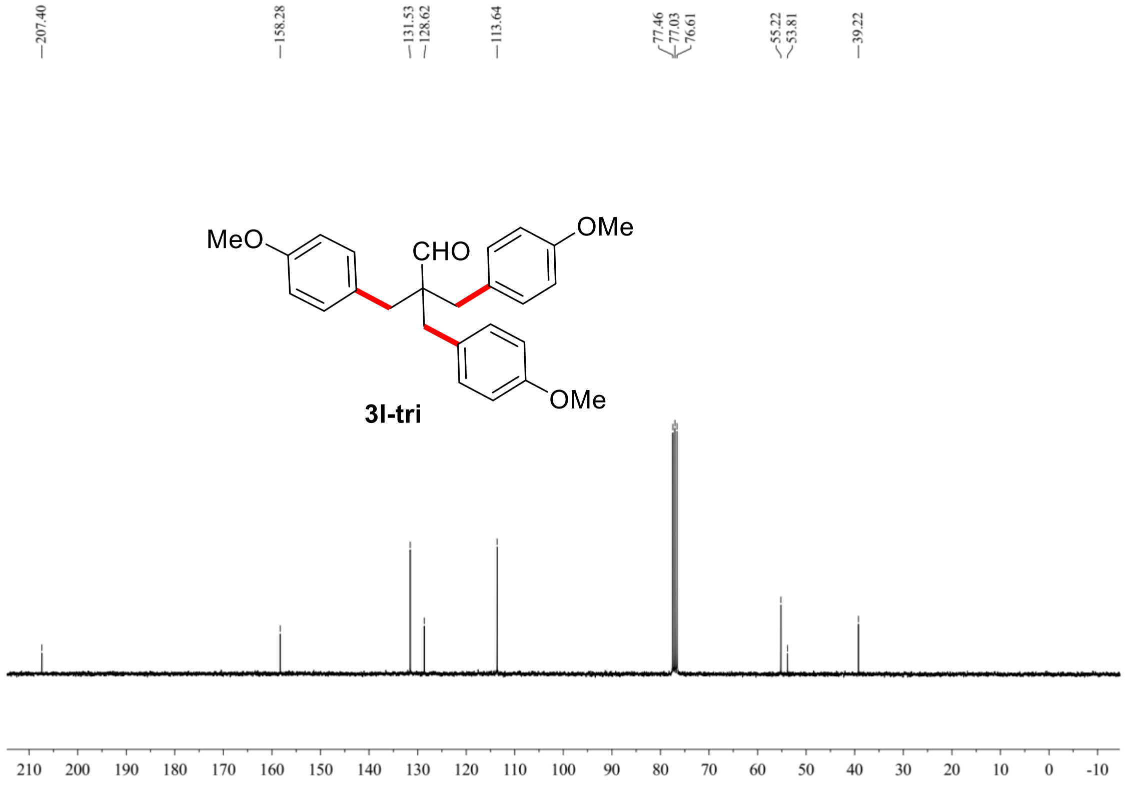

Figure S47 ^{13}C NMR spectrum (75MHz, $CDCl_3$, 298K) of **3l-tri**

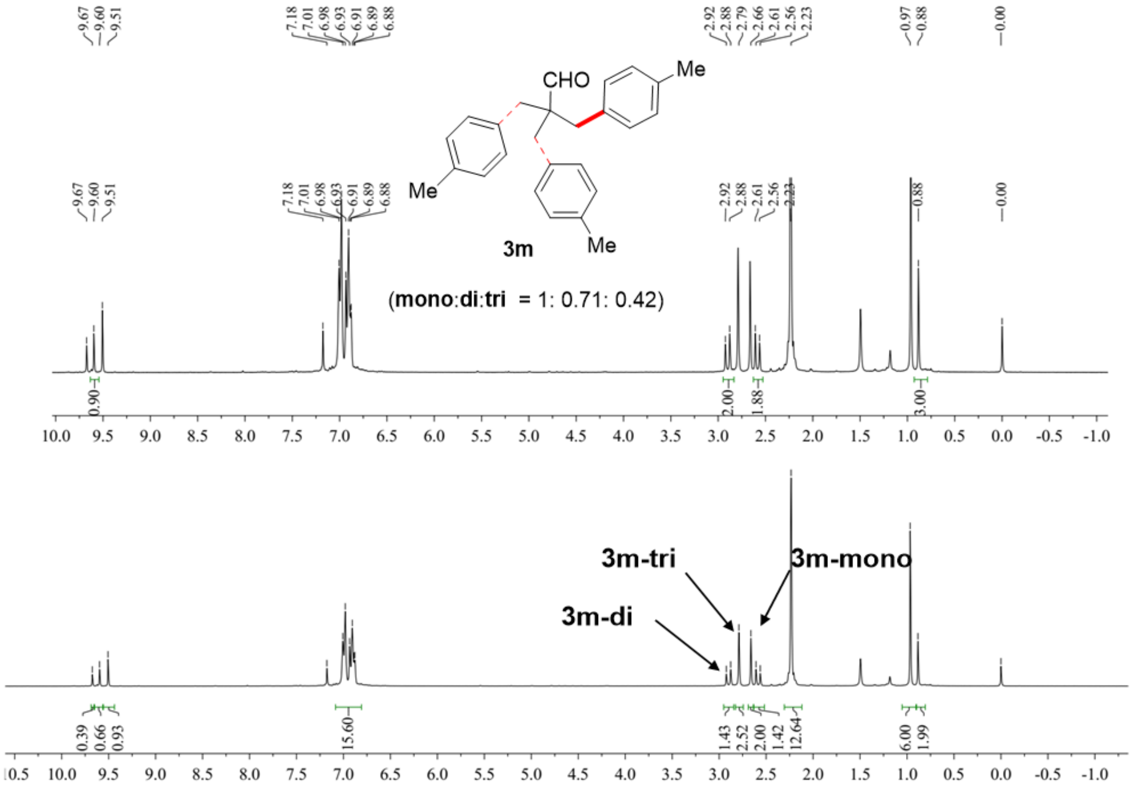

Figure S48 ^{1}H NMR spectrum (300MHz, $CDCl_3$, 298K) of **3m**

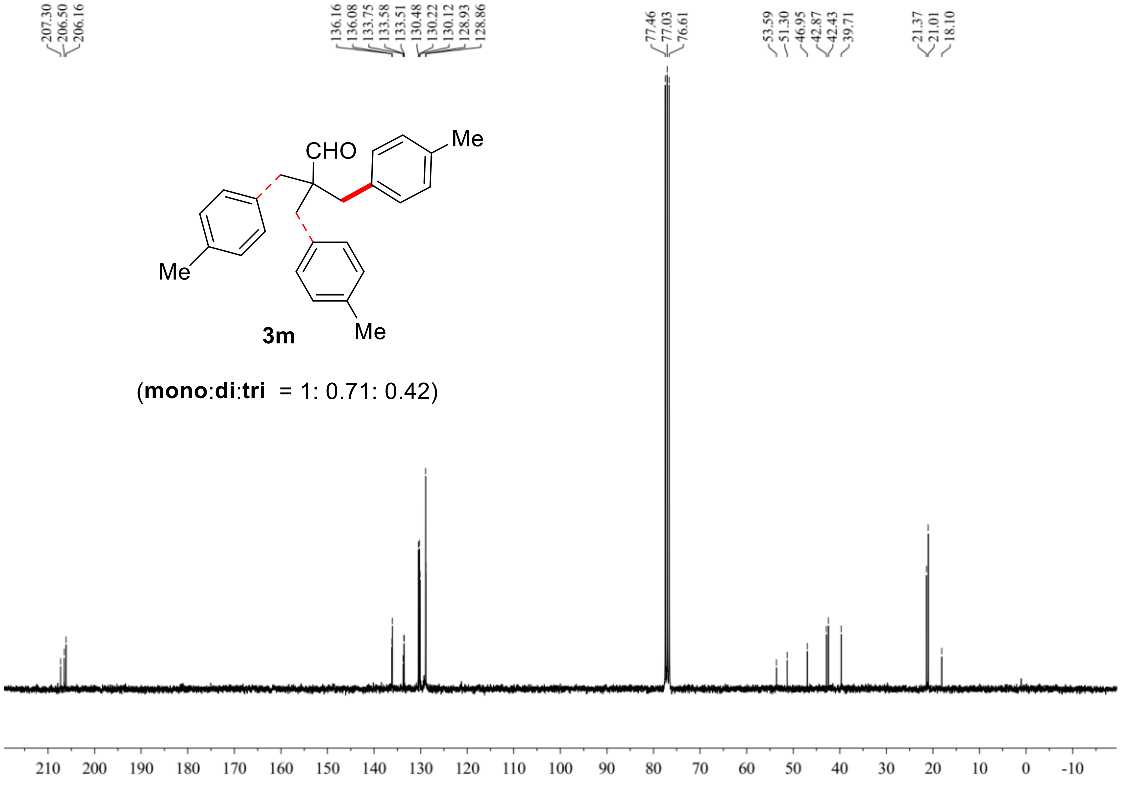

Figure S49 ^{13}C NMR spectrum (75MHz, $CDCl_3$, 298K) of **3m**

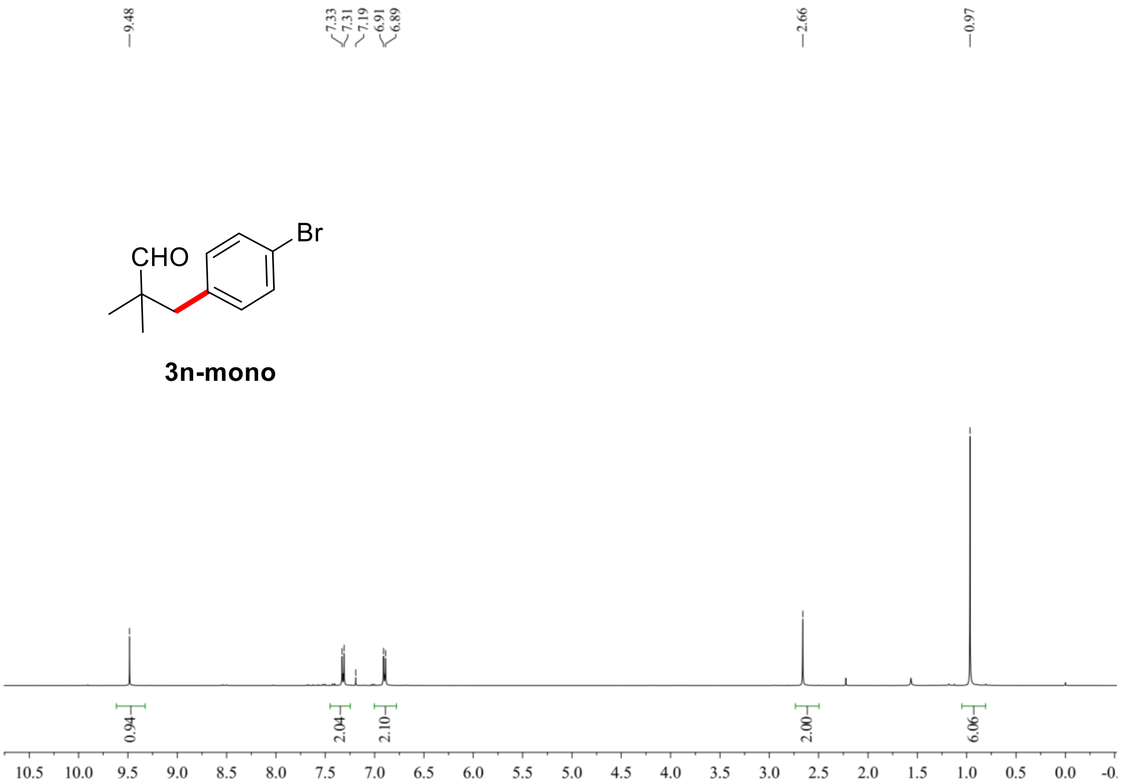

Figure S50 ^{1}H NMR spectrum (400MHz, $CDCl_3$, 298K) of **3n-mono**

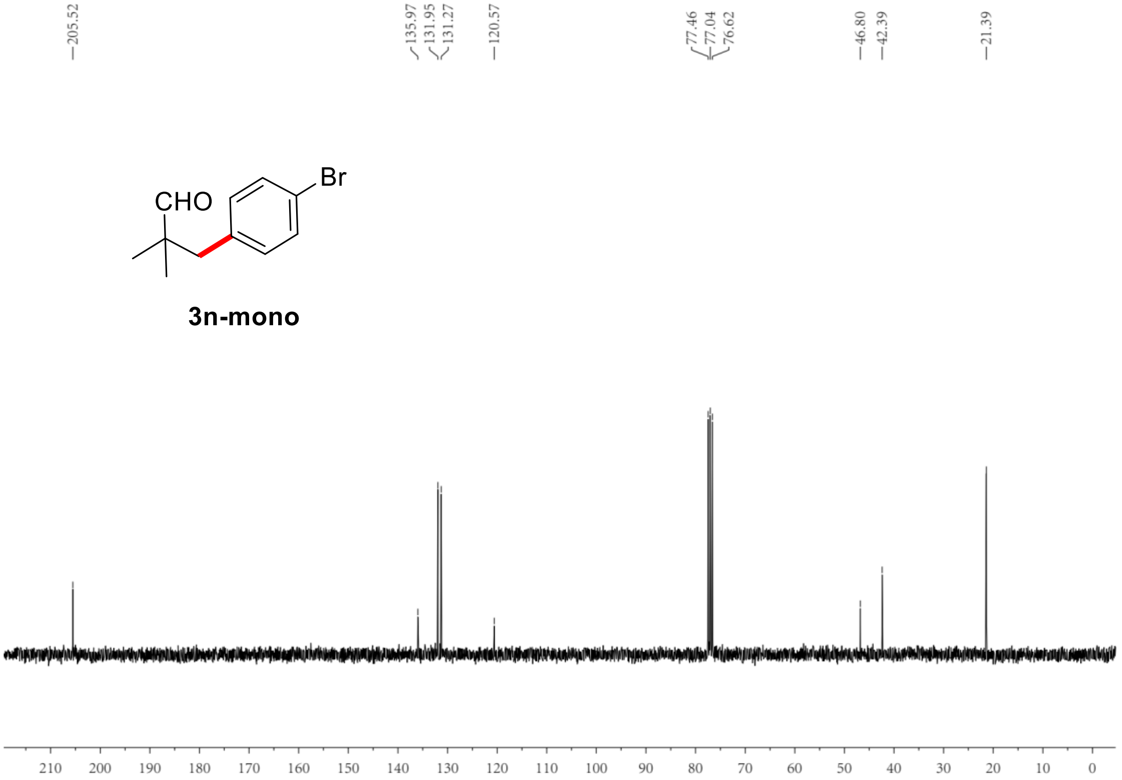

Figure S51 ^{13}C NMR spectrum (75MHz, $CDCl_3$, 298K) of **3n-mono**

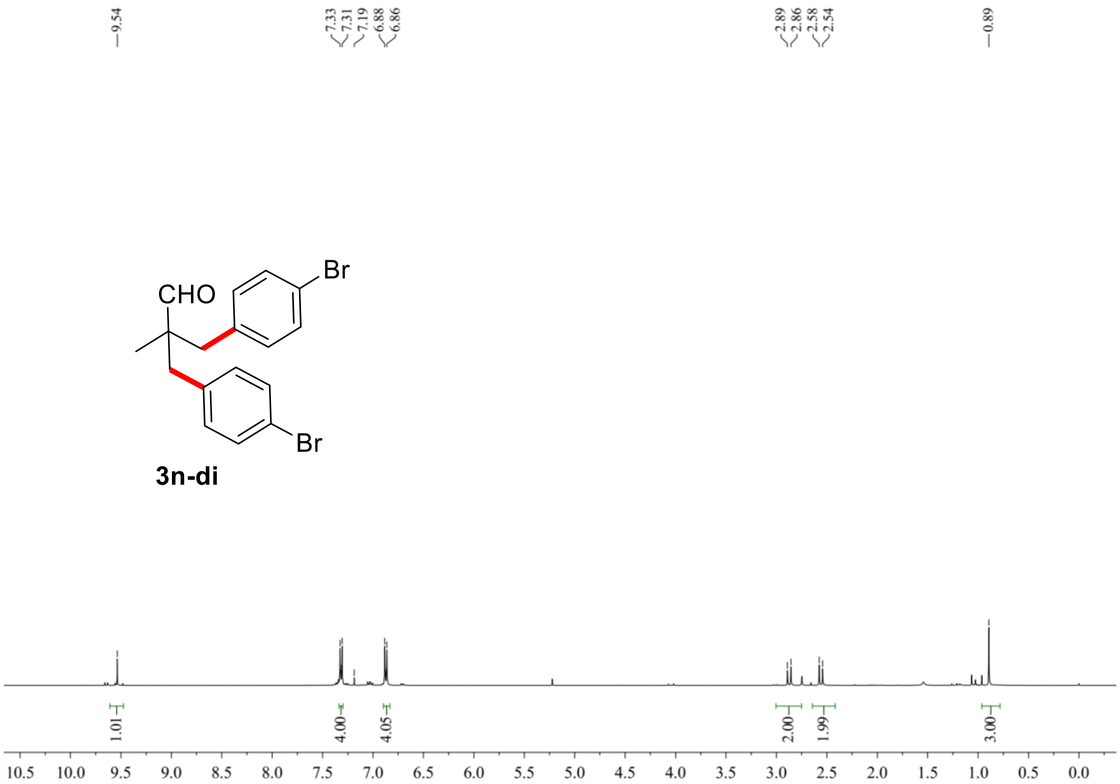

Figure S52 ¹H NMR spectrum (400MHz, $CDCl_3$, 298K) of **3n-di**

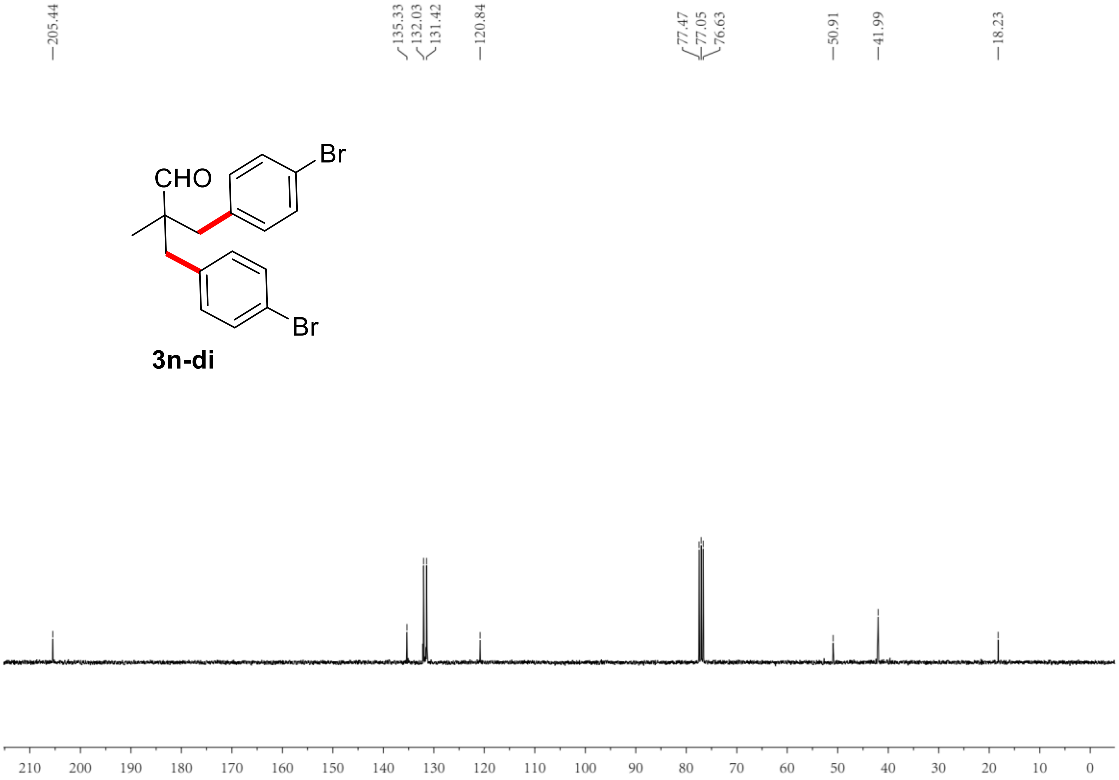

Figure S53 ¹³C NMR spectrum (75MHz, $CDCl_3$, 298K) of **3n-di**

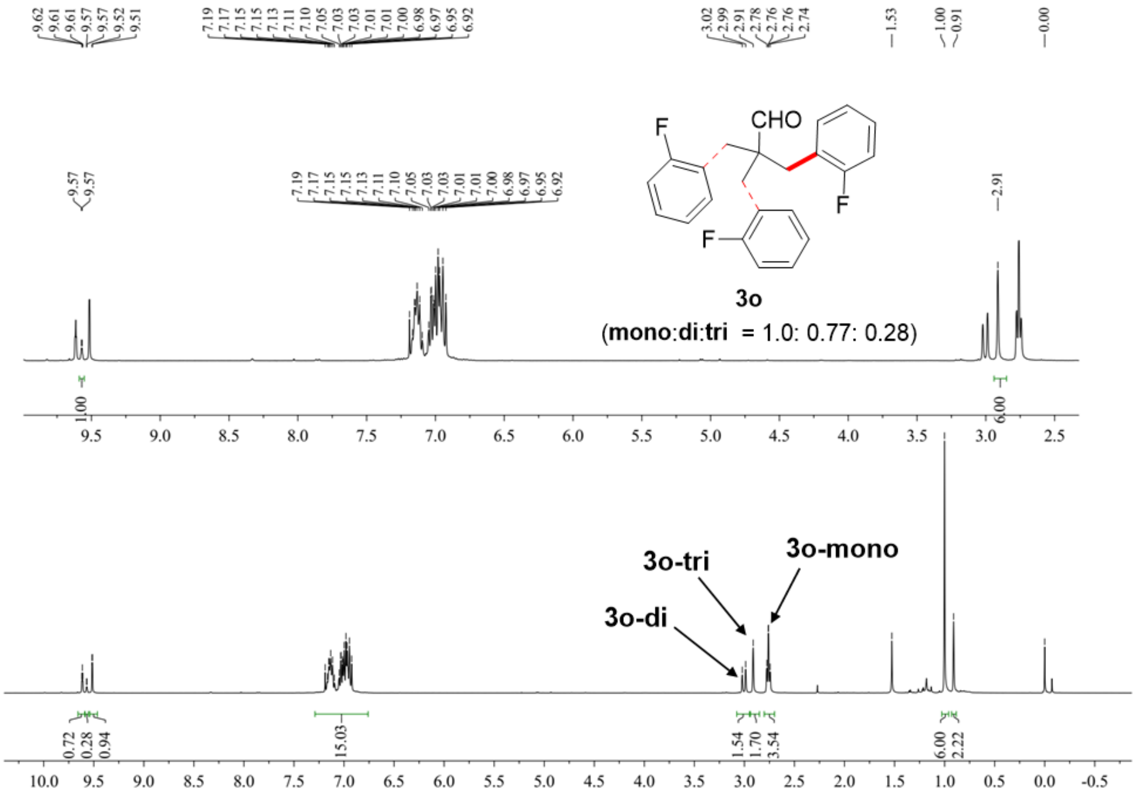

Figure S54 ^{1}H NMR spectrum (400MHz, $CDCl_3$, 298K) of **3o**

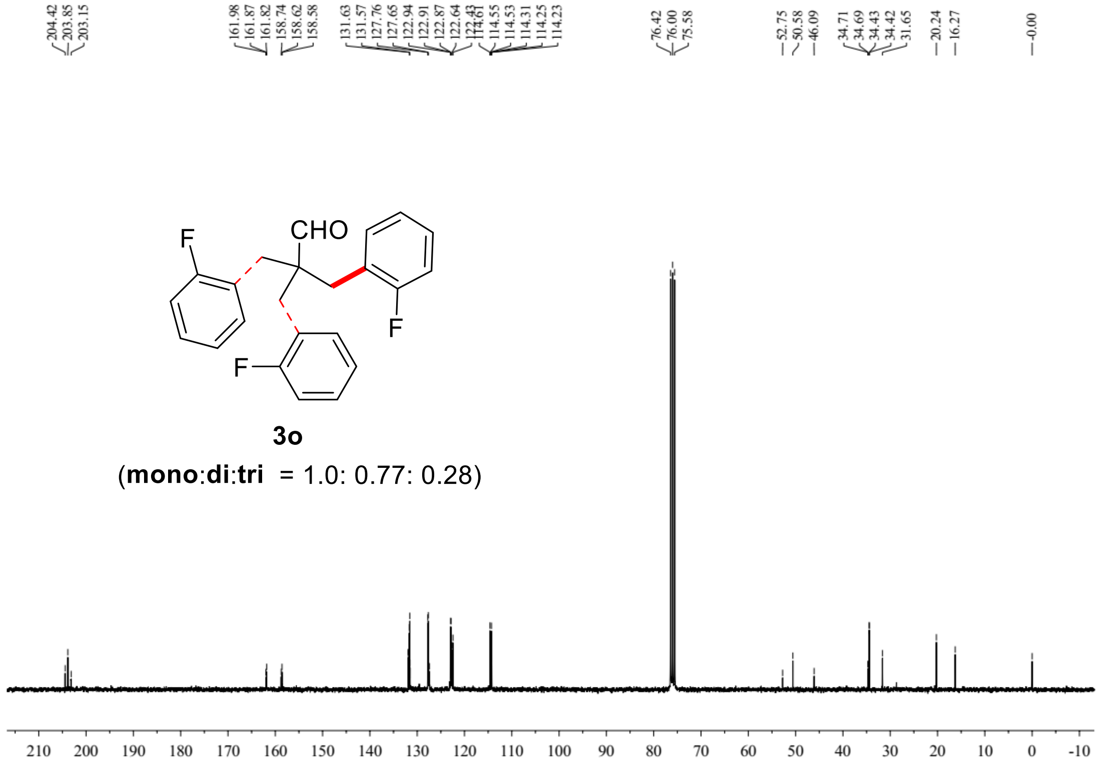

Figure S55 ^{13}C NMR spectrum (75MHz, $CDCl_3$, 298K) of **3o**

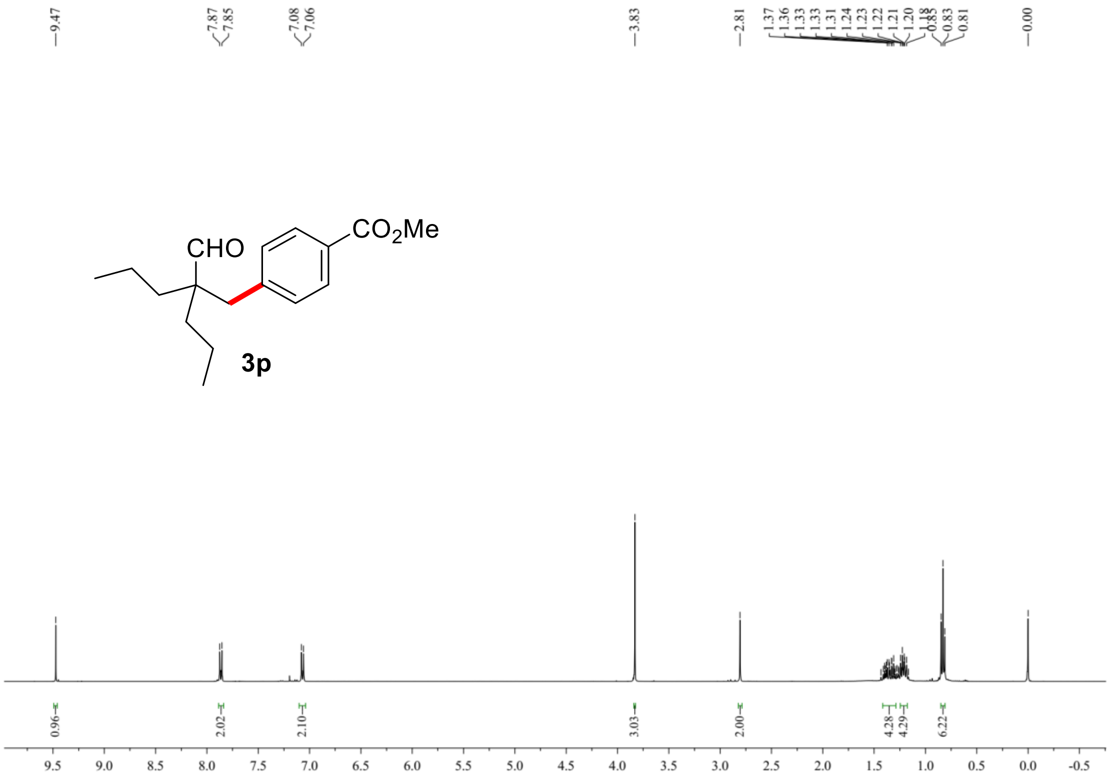

Figure S56 ¹H NMR spectrum (400MHz, $CDCl_3$, 298K) of **3p**

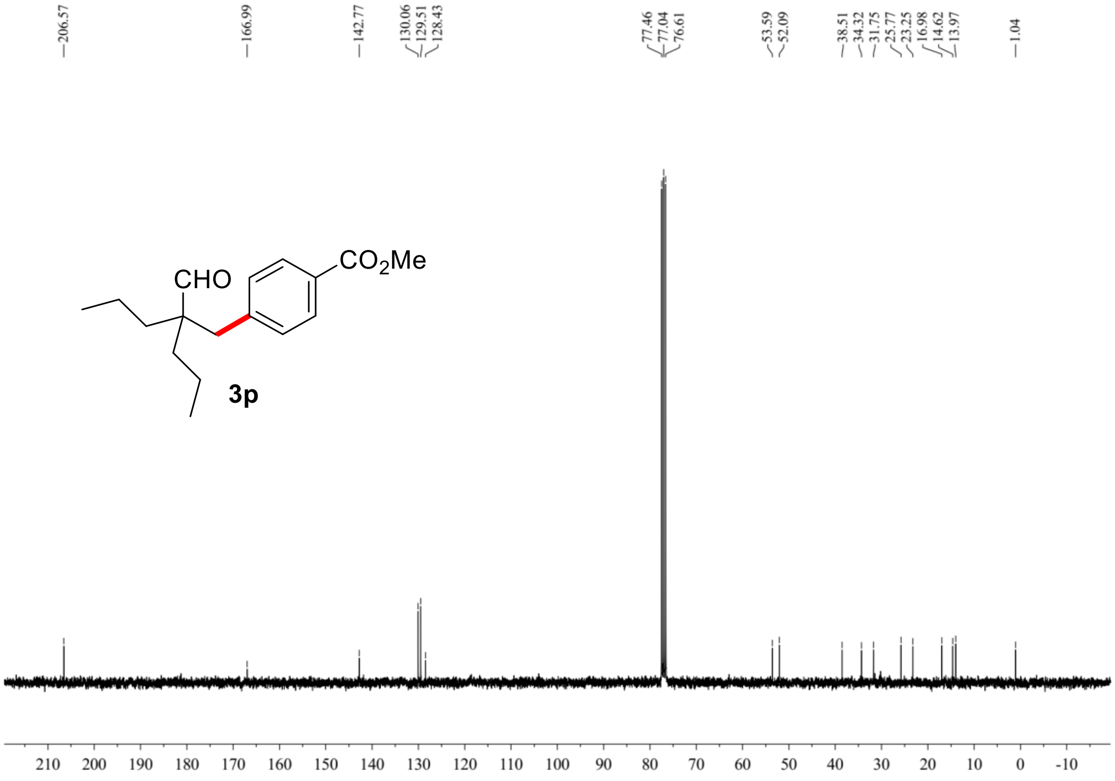

Figure S57 ¹³C NMR spectrum (75MHz, $CDCl_3$, 298K) of **3p**

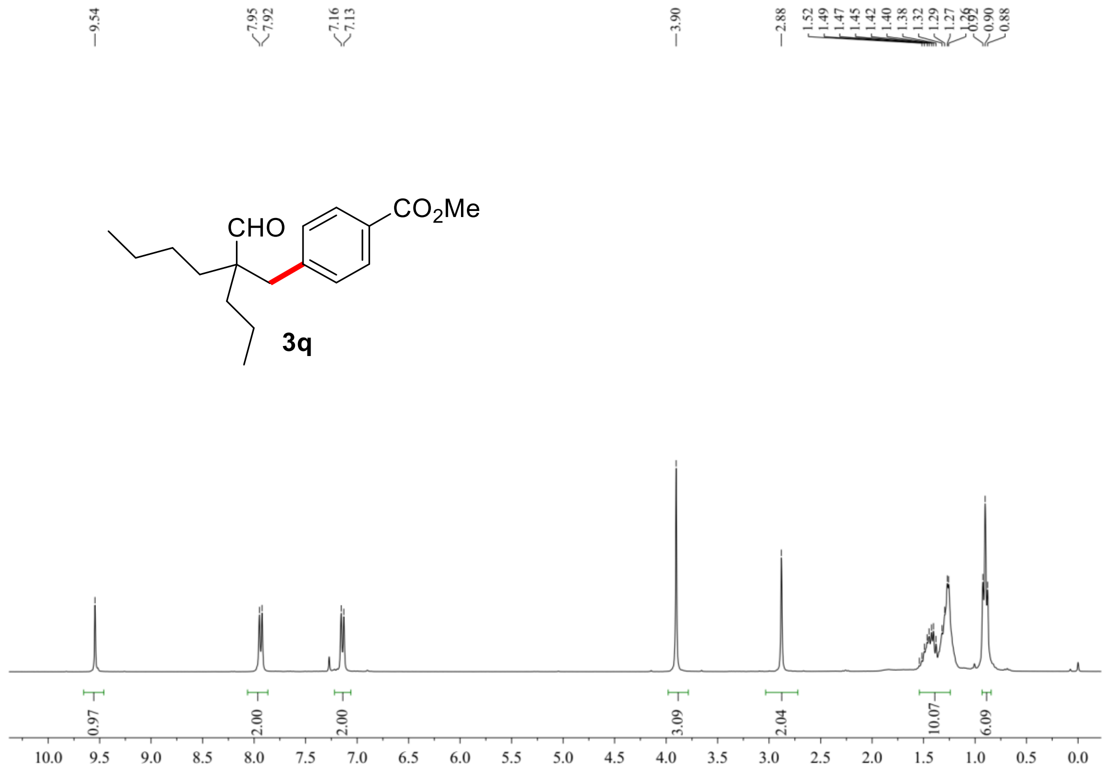

Figure S58 ^{1}H NMR spectrum (300MHz, $CDCl_3$, 298K) of **3q**

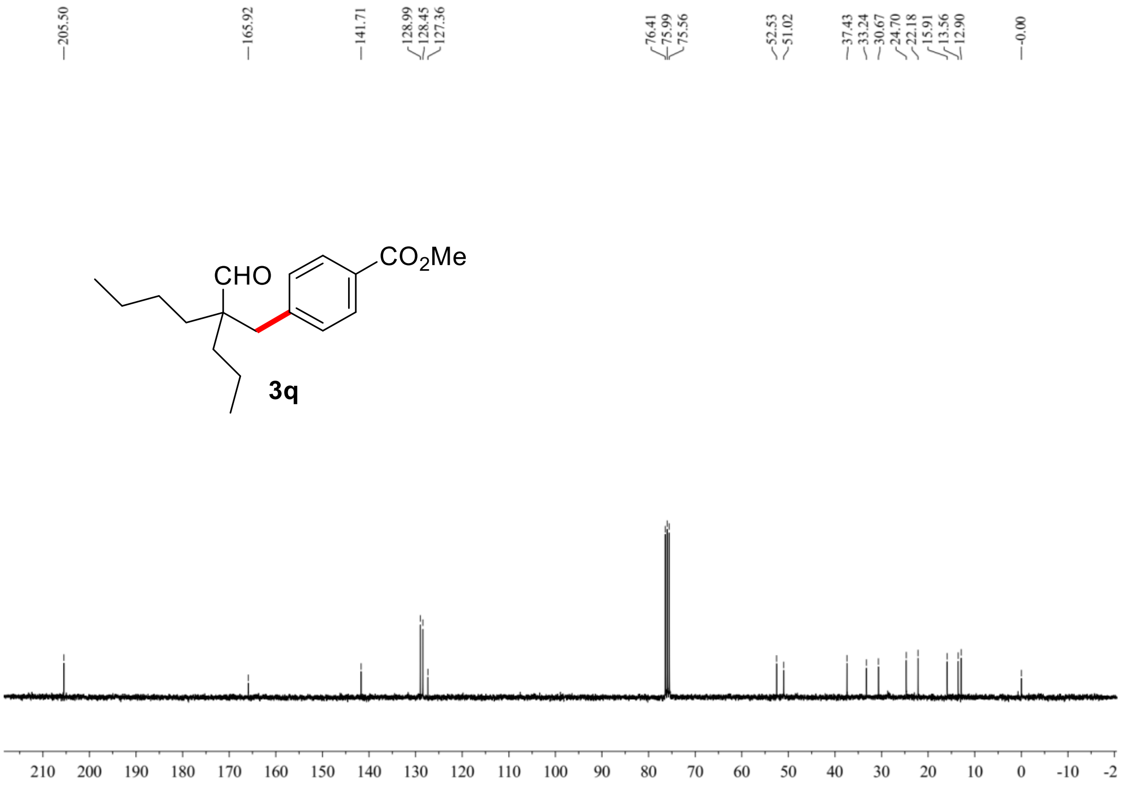

Figure S58 ^{13}C NMR spectrum (75MHz, $CDCl_3$, 298K) of **3q**

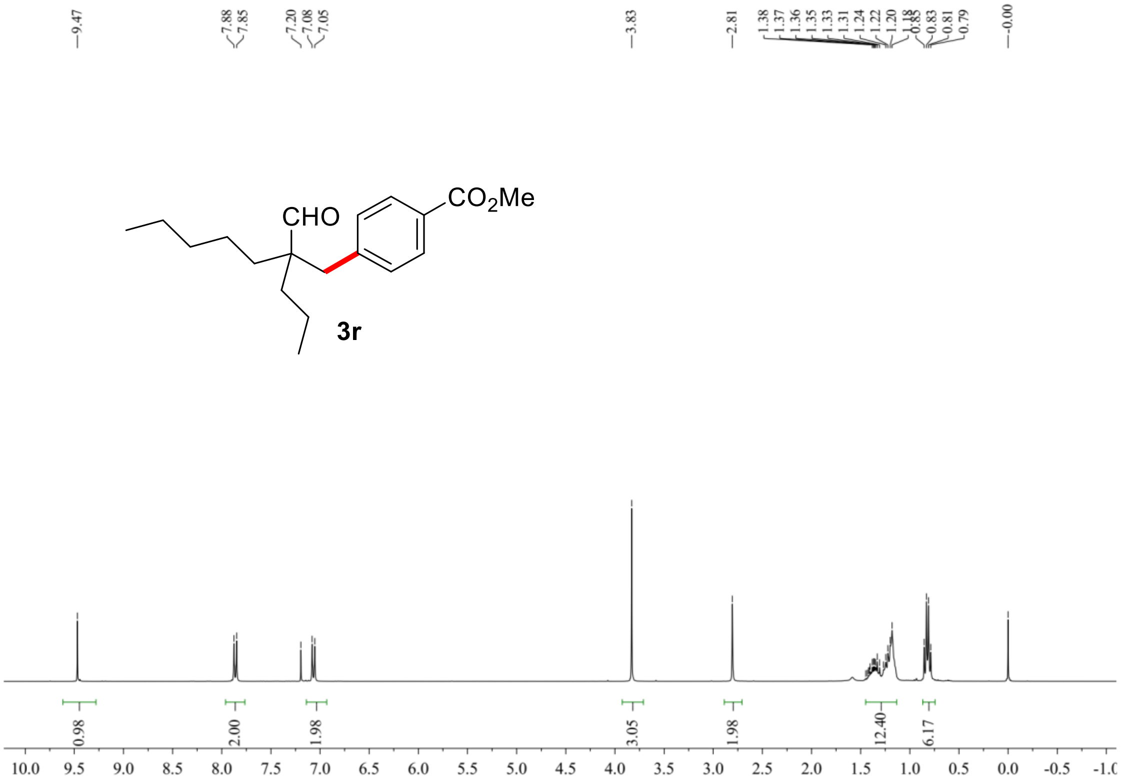

Figure S60 ¹H NMR spectrum (300MHz, $CDCl_3$, 298K) of **3r**

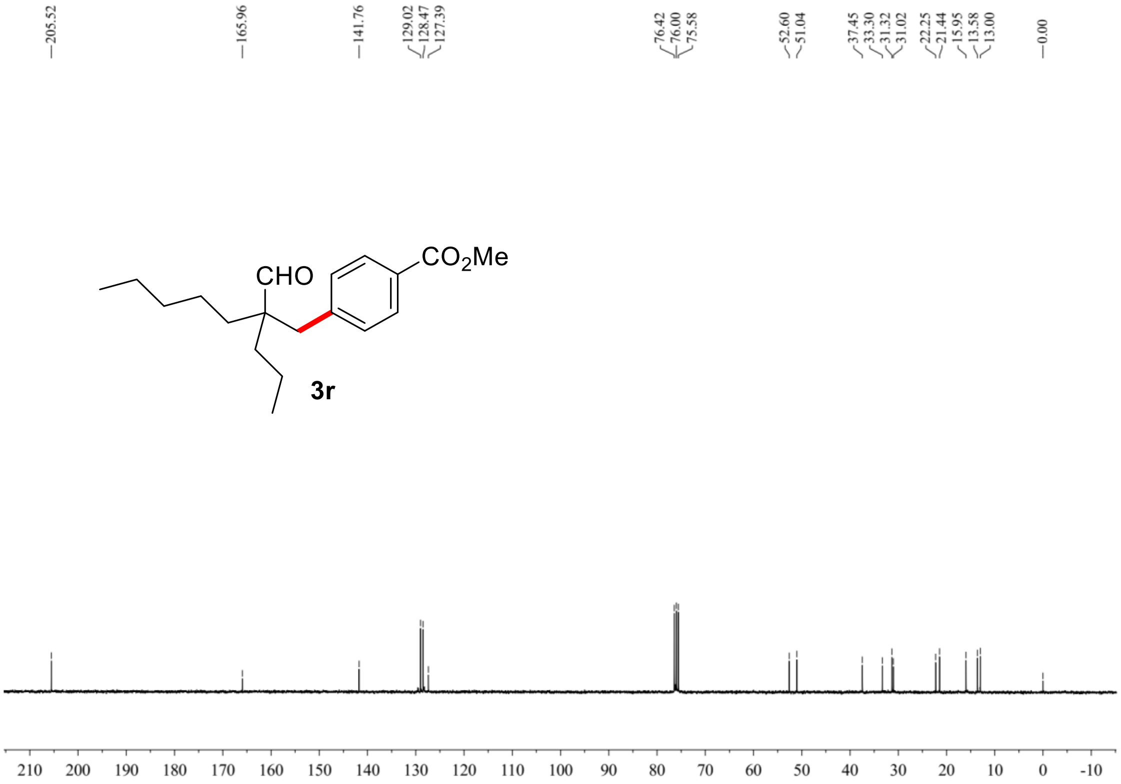

Figure S61 ¹³C NMR spectrum (75MHz, $CDCl_3$, 298K) of **3r**

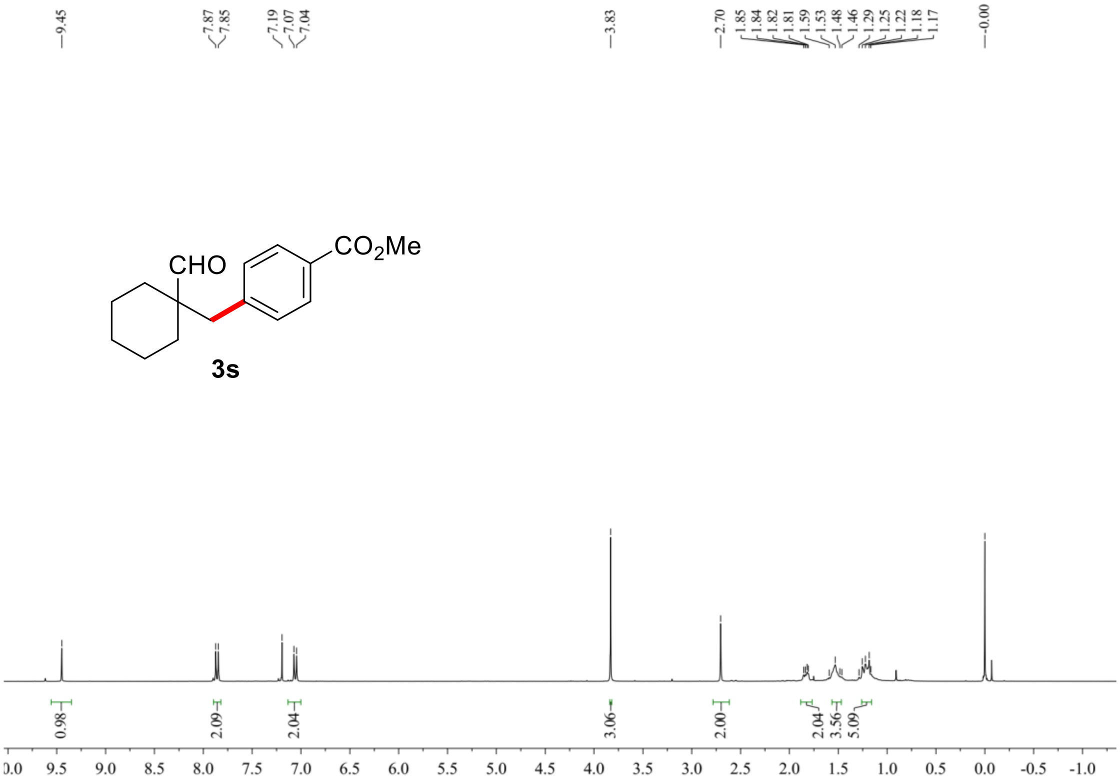

Figure S62 ^{1}H NMR spectrum (300MHz, CDCl3, 298K) of **3s**

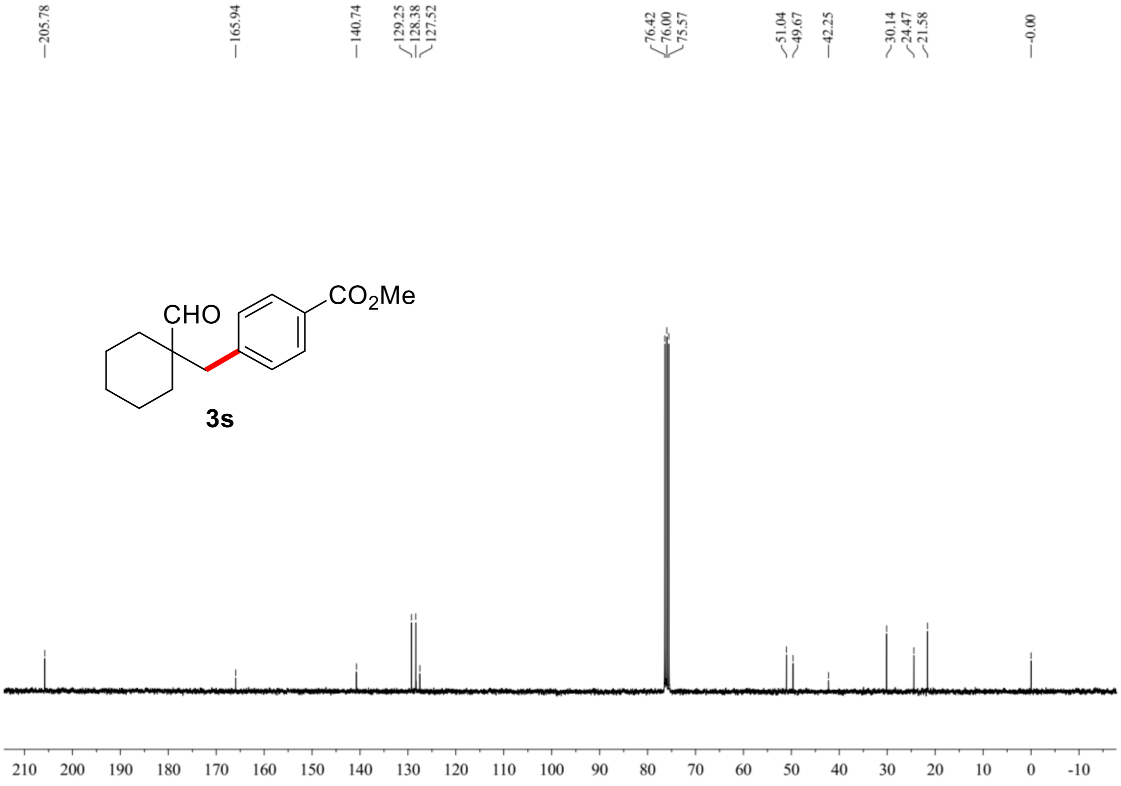

Figure S63 ^{13}C NMR spectrum (75MHz, CDCl3, 298K) of **3s**

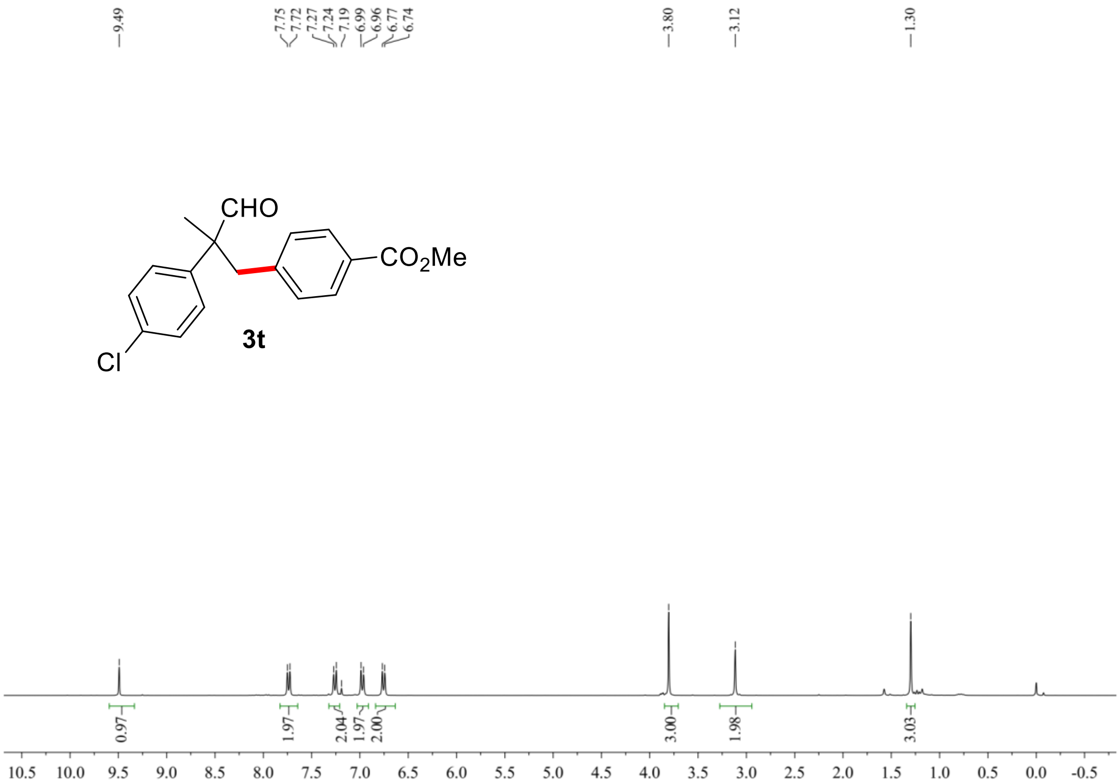

Figure S64 ^{1}H NMR spectrum (300MHz, $CDCl_3$, 298K) of **3t**

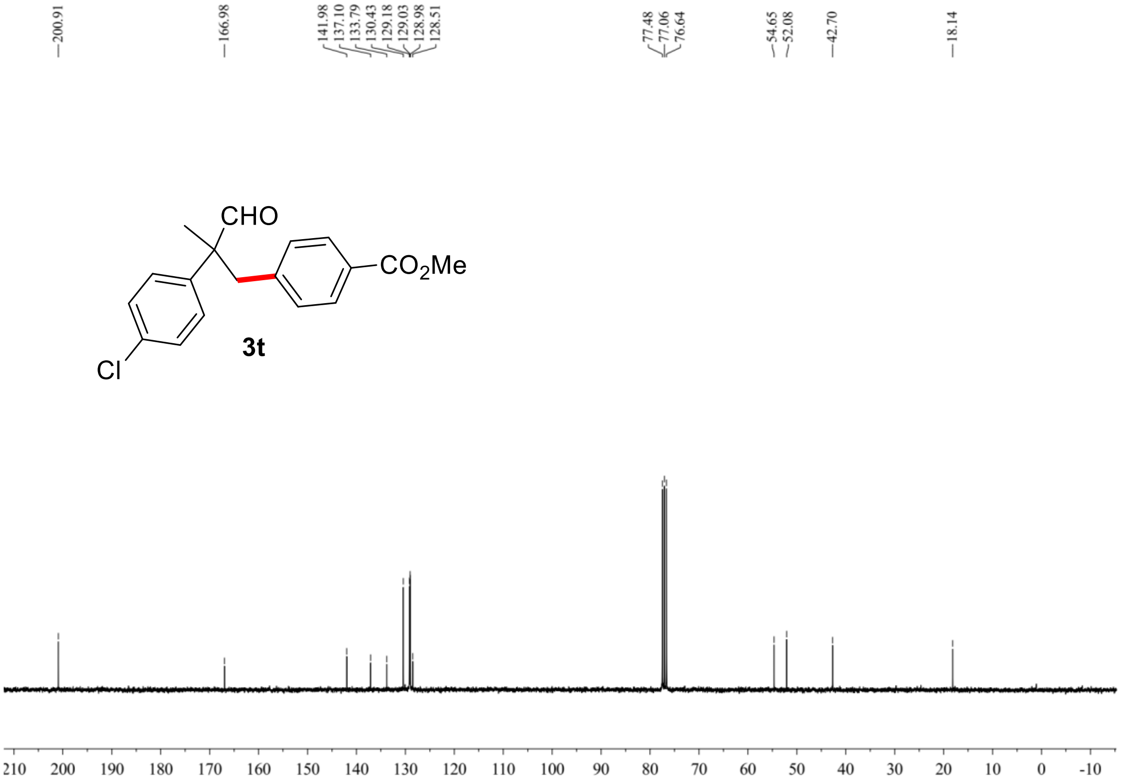

Figure S65 ^{13}C NMR spectrum (75MHz, $CDCl_3$, 298K) of **3t**

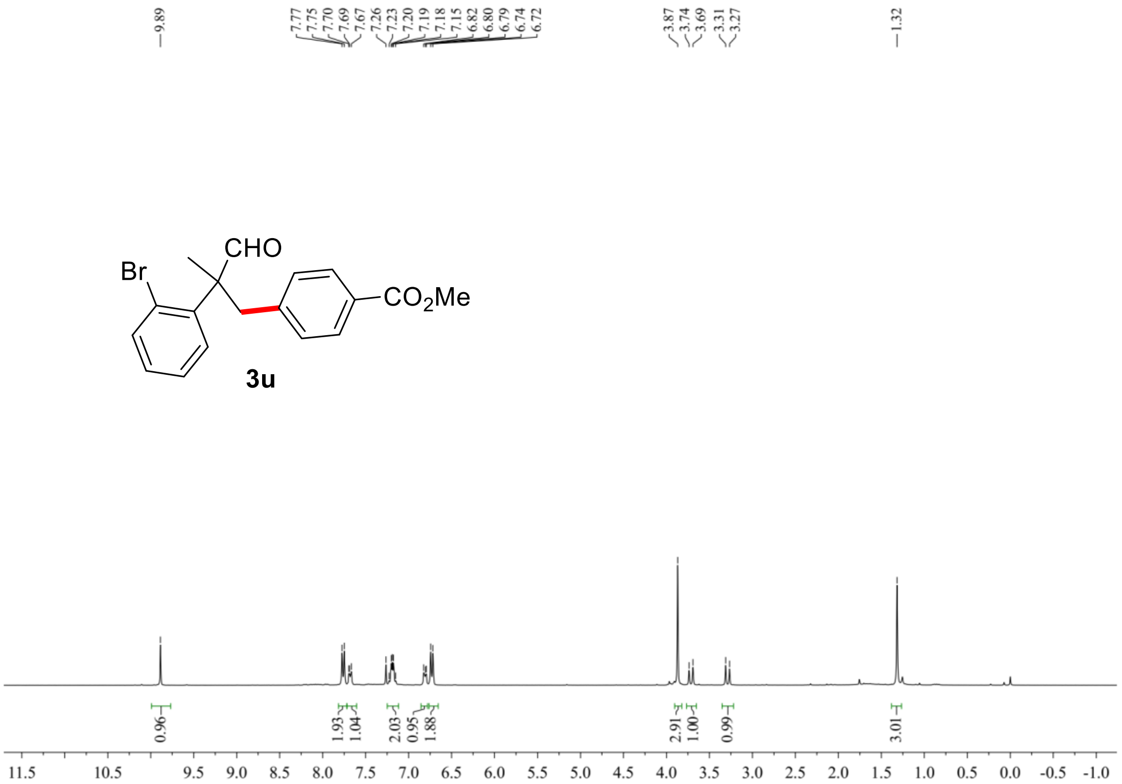

Figure S66 ^{1}H NMR spectrum (300MHz, $CDCl_3$, 298K) of **3u**

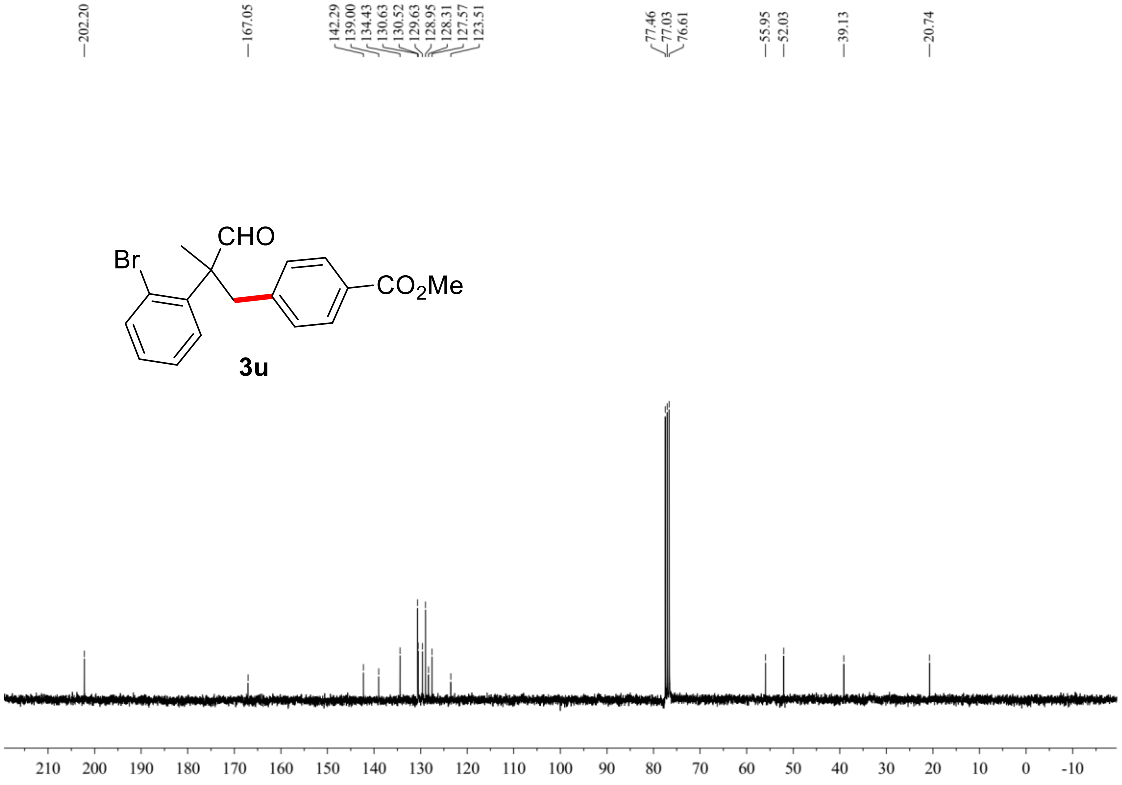

Figure S67 ^{13}C NMR spectrum (75MHz, $CDCl_3$, 298K) of **3u**

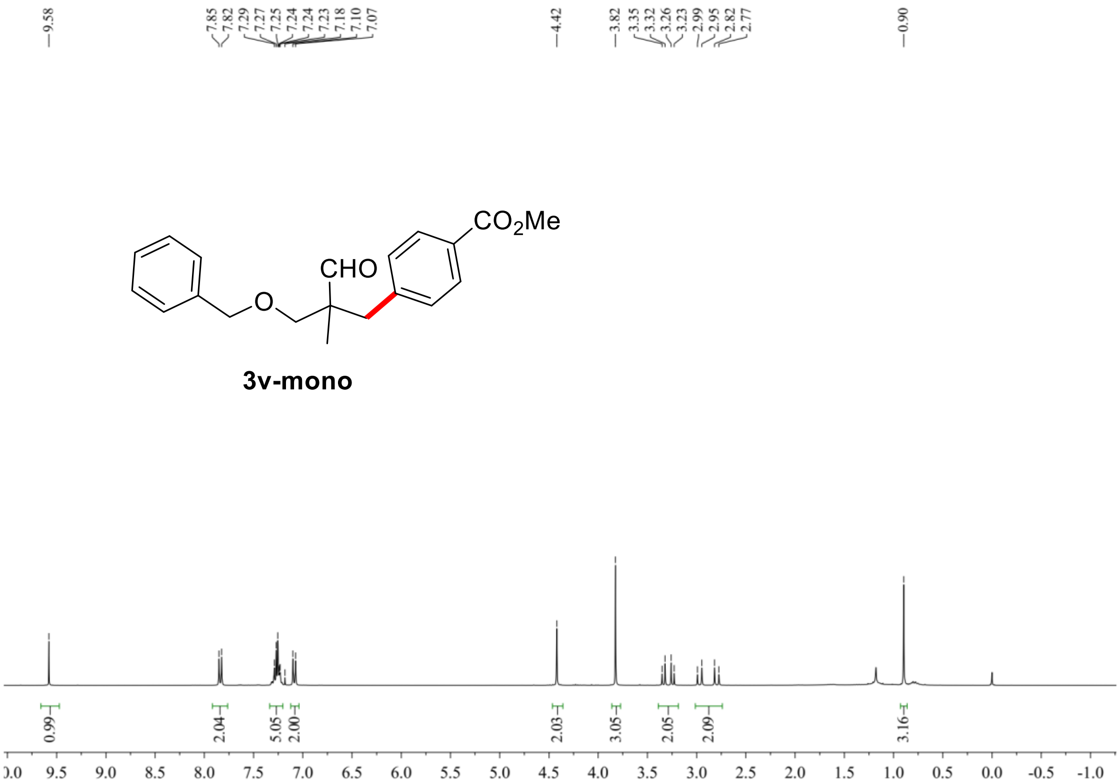

Figure S68 ^{1}H NMR spectrum (300MHz, $CDCl_3$, 298K) of **3v-mono**

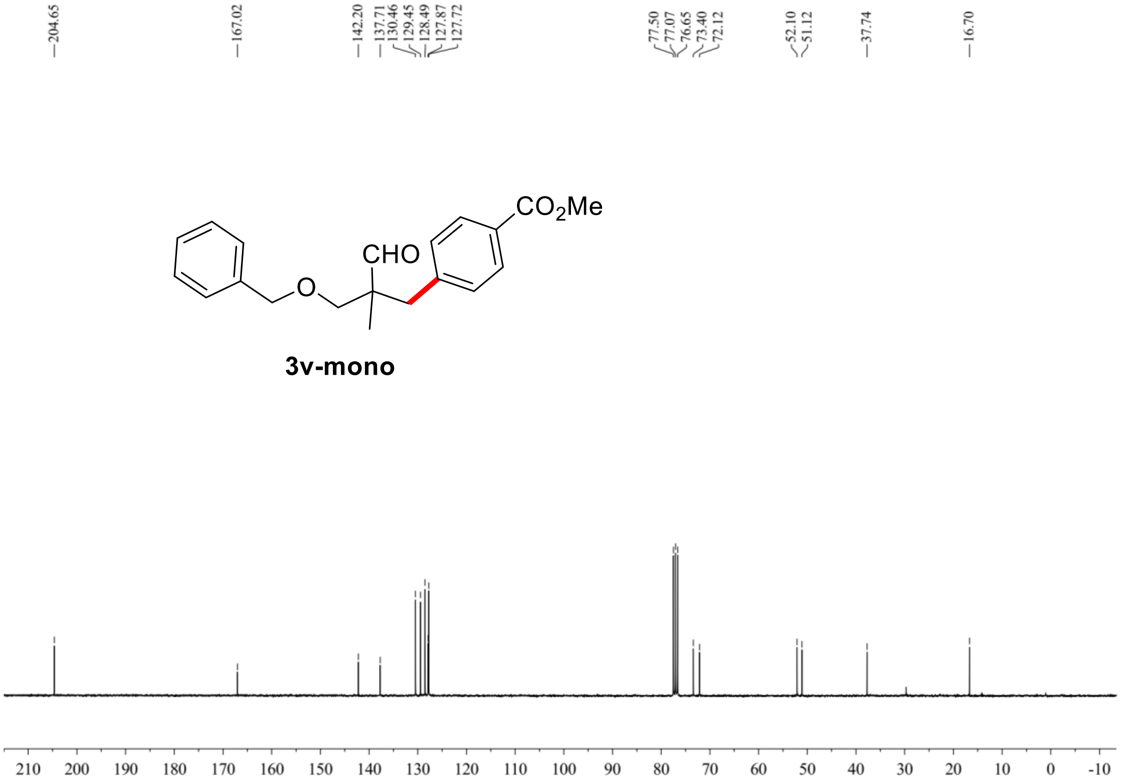

Figure S69 ^{13}C NMR spectrum (75MHz, $CDCl_3$, 298K) of **3v-mono**

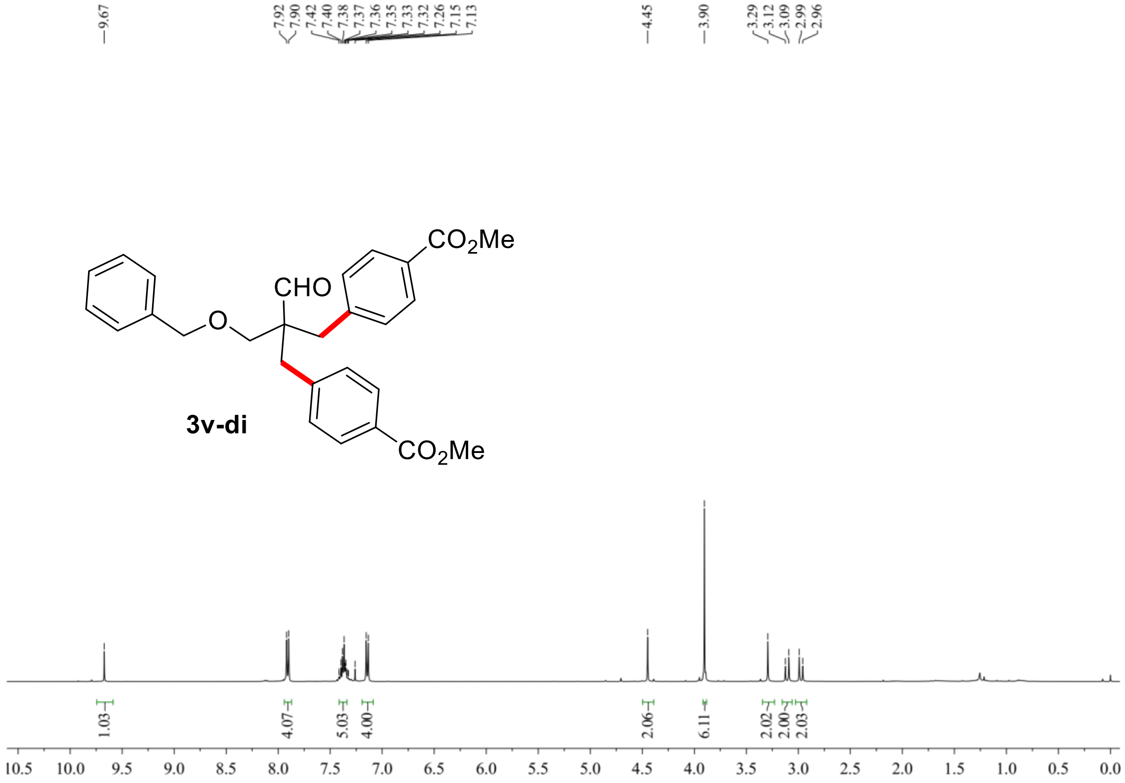

Figure S70 ^{1}H NMR spectrum (400MHz, $CDCl_3$, 298K) of **3v-di**

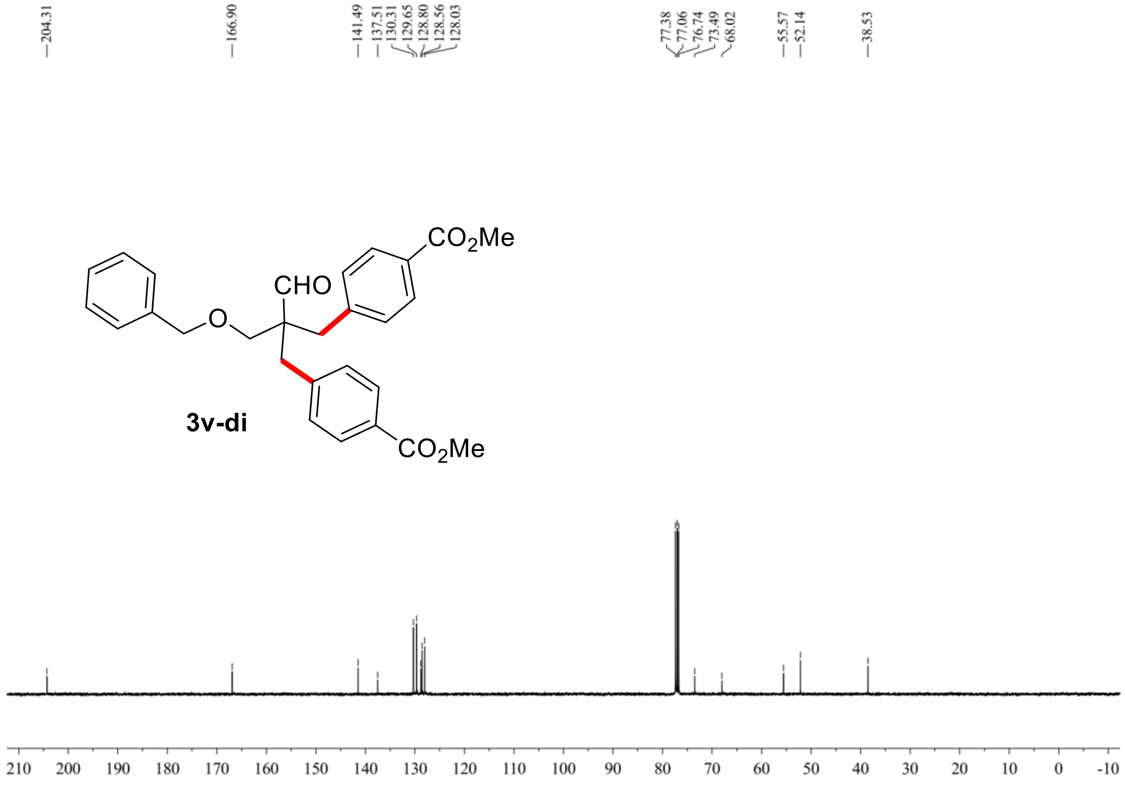

Figure S71 ^{13}C NMR spectrum (101MHz, $CDCl_3$, 298K) of **3v-di**

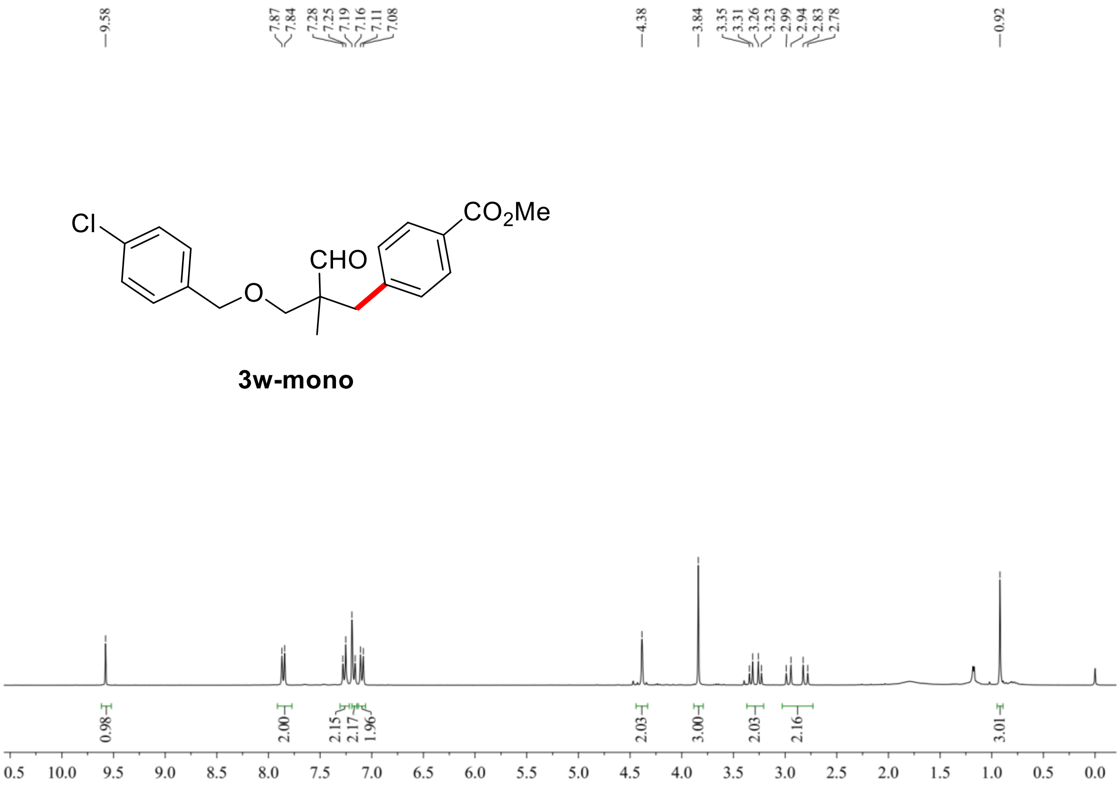

Figure S72 ^{1}H NMR spectrum (300MHz, $CDCl_3$, 298K) of **3w-mono**

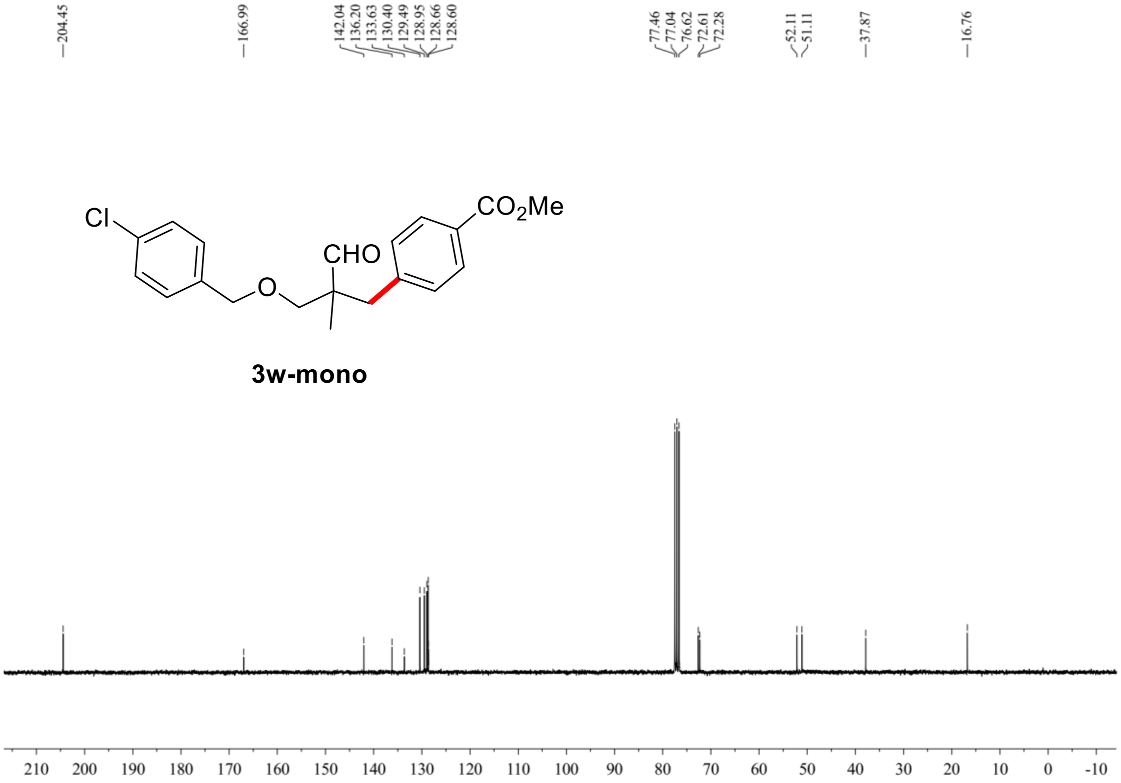

Figure S73 ^{13}C NMR spectrum (75MHz, $CDCl_3$, 298K) of **3w-mono**

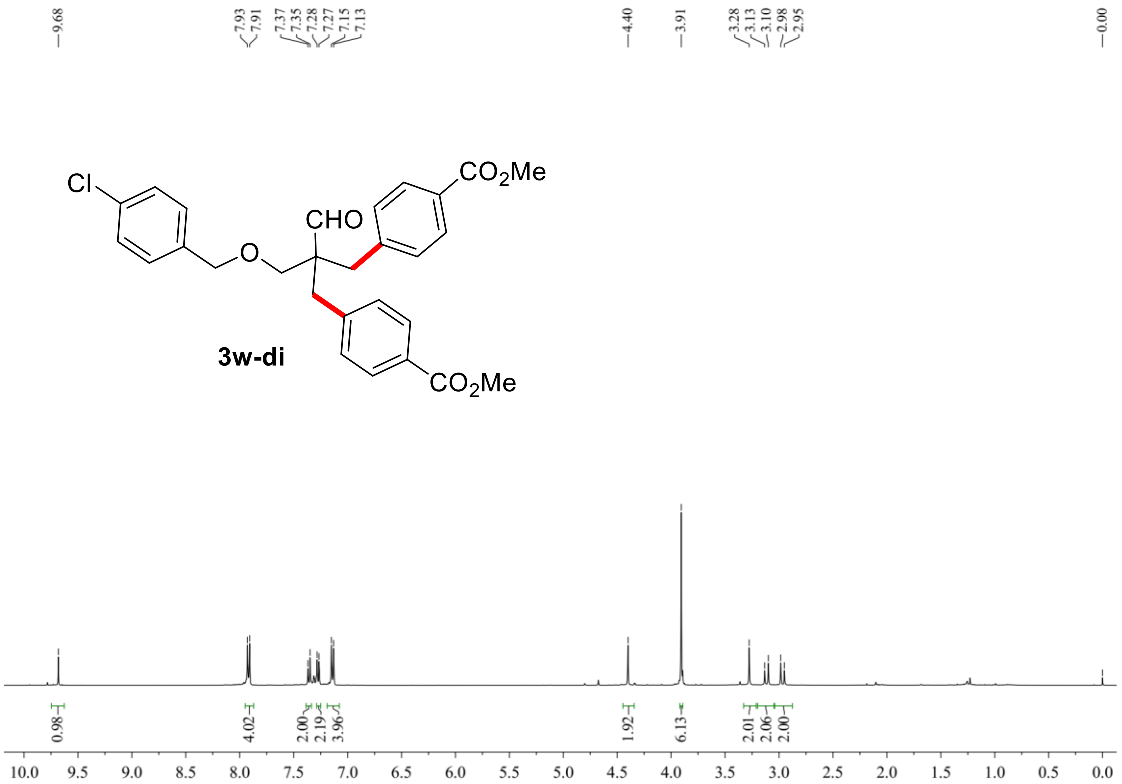

Figure S74 ^{1}H NMR spectrum (400MHz, $CDCl_3$, 298K) of **3w-di**

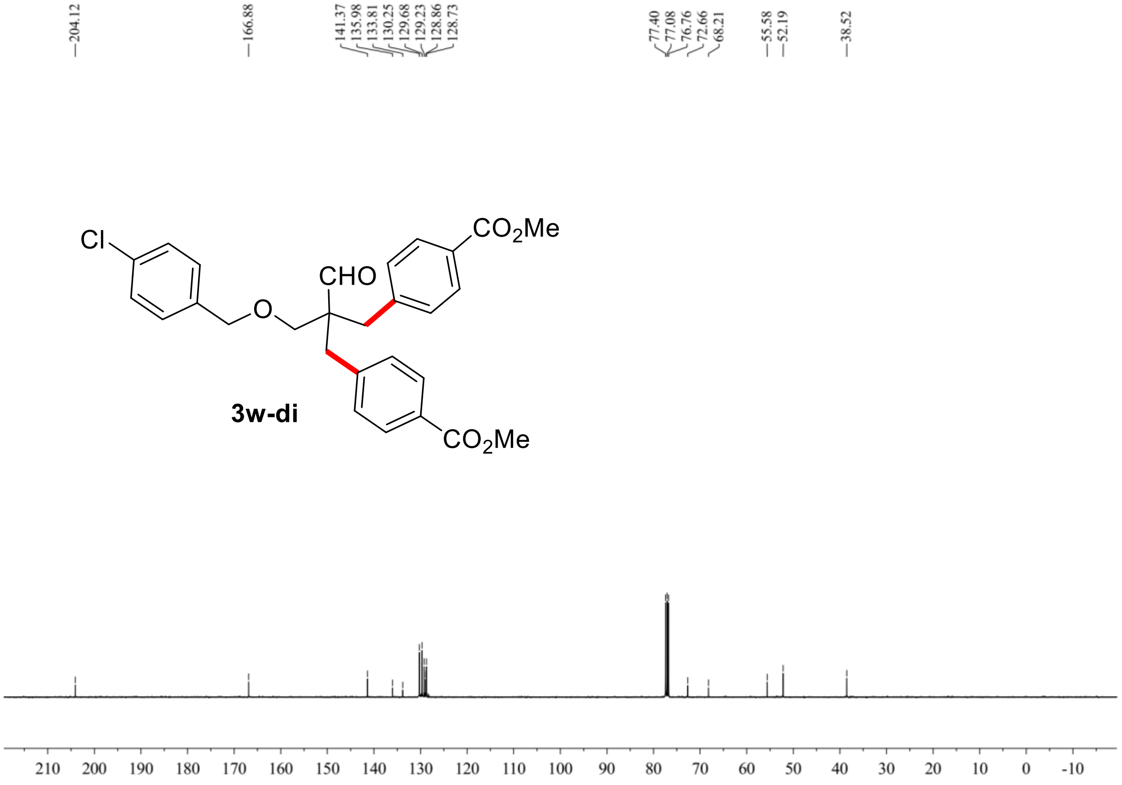

Figure S75 ^{1}H NMR spectrum (101MHz, $CDCl_3$, 298K) of **3w-di**
